# Muscarinic Acetylcholine Receptor Localization on Distinct Excitatory and Inhibitory Neurons Within the ACC and LPFC of the Rhesus Monkey

**DOI:** 10.3389/fncir.2021.795325

**Published:** 2022-01-11

**Authors:** Alexandra Tsolias, Maria Medalla

**Affiliations:** ^1^Department of Anatomy & Neurobiology, Boston University School of Medicine, Boston, MA, United States; ^2^Center for Systems Neuroscience, Boston University, Boston, MA, United States

**Keywords:** acetylcholine, muscarinic receptors, prefrontal cortex, non-human primate, inhibitory neurons, microcircuit

## Abstract

Acetylcholine (ACh) can act on pre- and post-synaptic muscarinic receptors (mAChR) in the cortex to influence a myriad of cognitive processes. Two functionally-distinct regions of the prefrontal cortex—the lateral prefrontal cortex (LPFC) and the anterior cingulate cortex (ACC)—are differentially innervated by ascending cholinergic pathways yet, the nature and organization of prefrontal-cholinergic circuitry in primates are not well understood. Using multi-channel immunohistochemical labeling and high-resolution microscopy, we found regional and laminar differences in the subcellular localization and the densities of excitatory and inhibitory subpopulations expressing m1 and m2 muscarinic receptors, the two predominant cortical mAChR subtypes, in the supragranular layers of LPFC and ACC in rhesus monkeys (*Macaca mulatta*). The subset of m1^+^/m2^+^ expressing SMI-32^+^ pyramidal neurons labeled in layer 3 (L3) was denser in LPFC than in ACC, while m1^+^/m2^+^ SMI-32^+^ neurons co-expressing the calcium-binding protein, calbindin (CB) was greater in ACC. Further, we found between-area differences in laminar m1^+^ dendritic expression, and m2^+^ presynaptic localization on cortico-cortical (VGLUT1^+^) and sub-cortical inputs (VGLUT2^+^), suggesting differential cholinergic modulation of top-down vs. bottom-up inputs in the two areas. While almost all inhibitory interneurons—identified by their expression of parvalbumin (PV^+^), CB^+^, and calretinin (CR^+^)—expressed m1^+^, the localization of m2^+^ differed by subtype and area. The ACC exhibited a greater proportion of m2^+^ inhibitory neurons compared to the LPFC and had a greater density of presynaptic m2^+^ localized on inhibitory (VGAT^+^) inputs targeting proximal somatodendritic compartments and axon initial segments of L3 pyramidal neurons. These data suggest a greater capacity for m2^+^-mediated cholinergic suppression of inhibition in the ACC compared to the LPFC. The anatomical localization of muscarinic receptors on ACC and LPFC micro-circuits shown here contributes to our understanding of diverse cholinergic neuromodulation of functionally-distinct prefrontal areas involved in goal-directed behavior, and how these interactions maybe disrupted in neuropsychiatric and neurological conditions.

## Introduction

Ascending brainstem neuromodulatory inputs to the prefrontal cortex (PFC) play an important role in the control of arousal and motivation during executive function and decision making (Mesulam et al., 1984; Everitt and Robbins, 1997; Picciotto et al., 2012). Acetylcholine (ACh), one such neuromodulator, plays a key role in both memory and emotional processing as well as PFC-mediated higher-order cognitive functions (Barbas, 2000; Hasselmo and Sarter, 2011). Behavioral studies have shown that cholinergic stimulation of the PFC enhanced cognitive performance in attention and working memory tasks in rodents (Deutsch, 1971), rhesus monkeys (Taffe et al., 1999; Vijayraghavan et al., 2018; Vijayraghavan and Everling, 2021), and humans (Drachman and Leavitt, 1974; Broks et al., 1988). Studies in rhesus monkeys have also shown that cholinergic muscarinic receptor antagonist, scopolamine (Bartus and Johnson, 1976) or deafferentation of cholinergic inputs to PFC (Croxson et al., 2011) produced deficits in delayed-response working memory tasks. Conversely, procholinergic drugs such as cholinesterase inhibitors ameliorated cognitive deficits seen in neurodegenerative disorders (Hampel et al., 2018; Moss, 2020). While these studies highlight the importance of ACh on PFC-mediated executive functions, the cellular and molecular effects of ACh in functionally-distinct PFC areas in primates remain unclear.

In primates, two key components of the frontal executive network—the lateral prefrontal cortex (LPFC) and the anterior cingulate cortex (ACC)—are markedly distinct with regard to the patterns of cholinergic innervation (Mesulam et al., 1984; Ghashghaei and Barbas, 2001). Cholinergic projections are significantly denser in limbic cortices, such as the ACC, compared to the LPFC (Mash et al., 1988; Ghashghaei and Barbas, 2001). The medially-located ACC has robust connectivity with limbic structures, such as the amygdala and hippocampus, and is therefore critical for motivational processing and cognitive-emotional interactions (Devinsky et al., 1995; Miller and Cohen, 2001; Barbas and Zikopoulos, 2007). However, the effects of cholinergic modulation on *in vivo* neuronal activity in primates have mostly been studied in LPFC, which is involved in sensory-motor processing and maintenance of relevant information in working memory (Barbas, 2000; Levy and Goldman-Rakic, 2000). Systemic treatment with the cholinergic muscarinic antagonist, scopolamine, diminished delay-related neuronal firing in LPFC during working memory tasks (Zhou et al., 2011; Major et al., 2015). Iontophoretic application of ACh within LPFC frontal eye fields resulted in increased neuronal task-related firing rates of diverse cell types (Dasilva et al., 2019). While cholinergic pathways have been shown to differentially alter intrinsic neuronal excitability and synaptic signaling in specific areas, layers, and cell types in rodent cortices (Parikh et al., 2007; Obermayer et al., 2017), the distribution of cholinergic receptors on distinct cell types in the primate LPFC and ACC remains unknown.

Pyramidal neurons in supragranular layers 2–3 of the PFC are principally responsible for cortico-cortical communication underlying cognitive processing (Barbas, 1986; Gonzalez-Burgos et al., 2000; Constantinidis et al., 2001; Amatrudo et al., 2012). Our previous work has shown that layer 3 (L3) pyramidal neurons in LPFC and ACC differ significantly in their inhibitory inputs (Medalla et al., 2017). Local GABAergic inhibitory interneurons, which constitute ~20–30% of all cortical neurons (Dombrowski et al., 2001), consist of diverse subtypes identified by their expression of calcium-binding proteins, parvalbumin (PV), calbindin (CB), and calretinin (CR), in primates (DeFelipe, 1997; Markram et al., 2004). PV neurons confer strong inhibition by targeting the proximal dendrites, somata, or axon initial segments of other neurons (DeFelipe, 1997; Kawaguchi and Kubota, 1998). On the other hand, CB neurons preferentially synapse on distal dendrites and spines (DeFelipe, 1997), similar to somatostatin-expressing interneurons in rodents (Rogers, 1992; Rocco et al., 2016). CR neurons synapse on other inhibitory neurons and have a dis-inhibitory role (del Rio and DeFelipe, 1997), analogous to VIP-expressing interneurons in rodents (Rogers, 1992; Gabbott and Bacon, 1997; Rocco et al., 2016). The ACC and LPFC differ markedly in the distribution of these neurochemical interneuron classes (Dombrowski et al., 2001). The neuromodulation of excitatory:inhibitory (E:I) balance shape functional circuitry in these PFC areas during behavior.

In primate and rodent cortices, ACh acts mainly through volume transmission (Mrzljak et al., 1995; Descarries and Mechawar, 2000; Sarter et al., 2009) and binds to muscarinic or nicotinic receptors extra-synaptic to cholinergic synapses, which are localized within or near glutamatergic and GABAergic synaptic sites (Sarter et al., 2009; Colangelo et al., 2019). Metabotropic G-protein coupled muscarinic acetylcholine receptors (mAChR), m1 and m2, are the predominant subtypes in the rhesus monkey cortex (Mrzljak et al., 1993; Picciotto et al., 2012). The m1 receptor, the main subtype expressed in the primate PFC, exerts a depolarizing effect on excitatory and inhibitory neurons when bound to ACh, and is localized on dendrites and spines, post-synaptic to glutamatergic and GABAergic synapses (Mrzljak et al., 1993; Carr and Surmeier, 2007). By contrast, the m2 receptor is largely located pre-synaptically on cortical glutamatergic and GABAergic nerve terminals, suppressing neurotransmitter release (Mrzljak et al., 1993; Kimura and Baughman, 1997; Salgado et al., 2007). Thus, the subcellular localization of m1 or m2 receptors underlies the functional consequences of cholinergic activation by fine-tuning E:I circuitry and activity during information processing (Picciotto et al., 2012; Groleau et al., 2015). However, the receptor and cell-type specific effects of ACh in ACC and LPFC remain largely unknown. Iontophoresis of an m1 receptor agonist into LPFC suppressed neuronal activity and overstimulation of m1 disrupted rule representations during a working memory task (Vijayraghavan et al., 2018). However, in the same area, m1 mAChR blockade (Vijayraghavan et al., 2018) and muscarinic stimulation increased the firing of both broad spiking and narrow spiking cell types (Dasilva et al., 2019). While these *in vivo* data point to diverse effects of ACh on physiologically-distinct task-related cell types, the involvement of excitatory or inhibitory neuronal classes, which cannot be distinguished reliably *in vivo* (Lee et al., 2021), remain unclear. Because anatomical structure constrains function, the goal of the current study is to determine the extent to which m1 and m2 receptors are expressed across distinct excitatory and inhibitory subpopulations and subcellular compartments in the ACC and LPFC. The anatomical localization of muscarinic receptors on these micro-circuits contributes to our understanding of prefrontal cholinergic neuromodulation and how this maybe be disrupted in neuropsychiatric and neurological conditions.

## Materials and Methods

### Experimental Subjects

Brain tissue used in this study was obtained from six young rhesus monkeys of both sexes (*Macaca mulatta*; 9 ± 1.13 years; two females and four males) that were a part of a larger program of studies on brain aging and cognition. Monkeys were obtained from National Primate Centers and private vendors and housed individually in the Laboratory Animal Science Center at Boston University School of Medicine; the facilities are fully accredited by the Association for Assessment and Accreditation of Laboratory Animal Care, with animal research conducted in strict accordance with guidelines of the National Institutes of Health’s Guide for the Care and Use of Laboratory Animals and Public Health Service Policy on Humane Care and Use of Laboratory Animals.

### Perfusion and Preparation of Tissue

Tissue was harvested using our well-established two-stage perfusion protocol allowing for the harvest of both live tissue and fixed tissue (Amatrudo et al., 2012) for parallel experiments not in the present study. After sedation with ketamine hydrochloride (10 mg/kg) the monkeys were deeply anesthetized with sodium pentobarbital (to effect, 15 mg/kg, i.v), and then perfused through the ascending aorta with ice-cold Krebs–Henseleit buffer containing (in mM): 6.4 Na_2_HPO_4_, 1.4 Na_2_PO_4_, 137 NaCl, 2.7 KCl, 5 glucose, 0.3 CaCl_2_, and 1 MgCl_2_, pH 7.4 (Sigma-Aldrich). Fresh tissues were collected from the left hemisphere for parallel biochemical and electrophysiological studies not in the present study. Once the fresh tissue harvest was complete, the perfusate was switched to 4% paraformaldehyde in 0.1M phosphate buffer (PB, ph 7.4, at 37°C) to fix the remaining whole brain. The fixed brain sample was blocked, *in situ*, in the coronal plane, removed from the skull, cryoprotected in a series of glycerol solutions, and flash-frozen in −70°C isopentane (Rosene et al., 1986). The brain was cut on a freezing microtome in the coronal plane at 30 or 60 μm and stored in cryoprotectant (15% glycerol, in 0.1M PB, pH 7.4) at −80°C (Estrada et al., 2017).

### Tissue Processing and Immunohistochemical Labeling for Fluorescent Microscopy

To visualize the distribution and extent of colocalization of m1 and m2 with distinct interneurons, pyramidal neurons, and inhibitory or excitatory vesicular transporter proteins, we batch processed 2–3 serial coronal 30 μm tissue sections through the ACC and LPFC per case for immunolabeling experiments adapted from (Medalla et al., 2017). Free-floating tissue sections were first rinsed (3 × 10 min, 4°C) in 0.01 M phosphate-buffered saline (PBS) and incubated in 50 mM glycine for 1 h at 4°C. Sections were then rinsed in 0.01 M PBS (3 × 10 min, 4°C), and antigen retrieval was performed with 10 mM sodium citrate (pH 8.5) in a 60–70°C water bath for 20 min. Sections were then rinsed in 0.01 M PBS (3 × 10 min, 4°C) and incubated in pre-block (0.01 M PBS, 5% bovine serum albumin (BSA), 5% normal donkey serum (NDS), 0.2% Triton X-100) to reduce any non-specific binding of secondary antibodies. Sections were incubated at 4°C for 48 h in a combination of primary antibodies to label distinct cell types with muscarinic receptors (see Table 1, diluted in 0.1 M PB, 0.2% acetylated BSA (BSA-c, Aurion), 1% NDS, 0.1% Triton X-100) as follows: (1) PV, CR, m1/m2; (2) CB, SMI-32, VGAT, m1/m2; (3) MAP2, VGAT, m1, m2; and (4) VGLUT1, VGLUT2, m2. To increase the penetration of the antibodies, two incubation sessions in a low-wattage microwave (2 × 10 min at 150 W) using the Pelco Biowave Pro (Ted Pella), followed by a 48-h incubation at 4°C with gentle agitation were employed. After rinsing (3 × 10 min) in 0.01 M PBS at 4°C, sections were incubated overnight in secondary antibodies diluted in incubation buffer (see Table 2) microwaved 2 × 10 min at 150 W (Ted Pella Pelco Biowave), and placed at 4°C for 24 h with gentle agitation. In some immunolabeling batches, biotinylated secondary antibodies and Streptavidin 546 conjugates were used to further amplify m2 labeling. Sections were then rinsed (3 × 10 min) in 0.1 M PB, mounted onto glass slides and cover-slipped with prolonged anti-fade gold mounting medium (ThermoFisher), and cured at room temperature in the dark. Control experiments performed included omitting the primary antibody or pre-absorbing the primary antibody with a control peptide were conducted and yielded no labeling. Additionally, density counts in the primary visual area (V1) yielded densities similar to those reported in (Disney and Aoki, 2008; data not shown).

**Table 1:** Primary antibodies utilized in immunohistochemistry.

**Primary Antibody**	**Host**	**Dilution**	**Vendor, Catalog #**	**RRID**
Calbindin D-28k (CB)	Rabbit	1:2,000	Swant, CB38	AB_10000340
Calretinin D-28K (CR)	Rabbit	1:2,000	Swant, 7697	AB_2721226
Parvalbumin (PV)	Guinea Pig	1:2,000	Swant, GP72	AB_2665495
Microtubule associated protein-2 (MAP2)	Chicken	1:1,000	Abcam, ab5392	AB_2138153
Neurofilament H non-phosphorylated (SMI32)	Mouse	1:2,000	BioLegend, 801701	AB_2564642
Muscarinic Receptor 1 (m1)	Goat	1:500	Abcam, ab77098	AB_1523990
Muscarinic Receptor 2 (m2)	Rat	1:500	Millipore, MAB367	AB_94952
Vesicular GABAergic Transporter (VGAT)	Guinea Pig	1:400	Synaptic Systems, 131004	AB_887873
Vesicular Glutamate Transporter (VGLUT1)	Rabbit	1:1,000	Synaptic Systems, 135303	AB_887875
Vesicular Glutamate Transporter (VGLUT2)	Guinea Pig	1:1,000	Synaptic Systems, 135404	AB_887884

**Table 2:** Secondary antibodies utilized in immunohistochemistry.

**Secondary antibody conjugate**	**Host-antigen**	**Dilution**	**Vendor, Catalog #**
Alexa 405	Donkey anti-mouse	1:200	ThermoFisher, A10036
Alexa 488	Donkey anti- Guinea Pig	1:200	Jackson, 706-545-148
Alexa 488	Donkey anti-chicken	1:200	Jackson, 703-545-155
Alexa 546	Donkey anti- Rabbit	1:200	Thermofisher, A10040
Alexa 647	Donkey anti- Guinea Pig	1:200	Jackson, 706-605-148
Alexa 633	Donkey anti-goat	1:200	Thermofisher, A21082
Alexa 647	Donkey anti-rat	1:200	Jackson, 712-606-150
Biotin	Donkey anti-rat	1:200	Jackson, 712-067-003
Streptavidin-546	Biotinylated Donkey anti-Rat	1:200	ThermoFisher, S11225

### Confocal Microscopy and Visualization of Immunofluorescent Labeling

Immunofluorescent labeling was imaged at high resolution using a laser-scanning confocal microscope (Leica SPE or Zeiss LSM 710). Image stacks were acquired using a plan apochromat 40×/1.3 NA oil-immersion objective at a resolution of 0.268 μm × 0.268 μm × 0.5 μm (Leica TCS SPE) or 0.208 × 0.208 × 0.5 μm (Zeiss LSM 710) voxel size. Based on architectonic maps of prefrontal cortices (Barbas and Pandya, 1989), medial area 24 of ACC and dorsal area 46 of LPFC were identified and imaged in a columnar fashion from the pial surface to the white matter boundary (three columns per area and case). The resulting image stacks were deconvolved to improve the signal-to-noise ratio and converted to 8-bit images for analysis using AutoQuant (Media Cybernetics).

### Interneuron and Pyramidal Cell Density Estimates

We quantified the density of immunolabeled somata of total mAChR m1^+^ and m2^+^ labeled cells and subpopulations of excitatory (MAP2^+^, SMI-32^+^) and inhibitory (CB^+^PV^+^CR^+^) neurons co-expressing m1^+^ and m2^+^ using adapted stereologic cell counting procedures (Fiala and Harris, 2001). The different cortical layers were delineated based on depth from the pial surface measured from matched Nissl sections. Fields imaged within layer 2 (L2) and layer 3 (L3) in each area were counted separately. Image stacks were deconvolved (AutoQuant, Media Cybernetics) and imported into FIJI^1^ (1997–2016). Each image acquired was a counting field of about 212.5 μm × 212.5 μm area and 18 μm in depth. To quantify the density of total single-labeled m1^+^ and m2^+^ cells, the individual m1 or m2 channels were extracted from each multi-channel image from five sets of immunolabeling experiments: (1) PV, CR, m1; (2) PV, CR, m2; (3) CB, SMI-32, VGAT, m1; (4) CB, SMI-32, VGAT, m2; and (5) MAP2, VGAT, m1, m2. Each single channel m1 or m2 image was taken as a sampling site, and cell densities across all images were averaged per case. Pyramidal vs. non-pyramidal neurons were identified based on morphology, as well as staining with MAP2^+^, SMI32^+^, and CB^+^. Morphologically identified pyramidal neurons were based on classic criteria, which included the pyramidal-shaped cell body and the prominent apical dendrite (Spruston, 2008). For CB^+^, non-pyramidal interneurons were distinguished morphologically and counted separately from CB^+^ pyramidal neurons. Immunolabeled cell bodies were manually counted using FIJI software and the “cell counter” plug-in marking single-labeled somata expressing particular excitatory and inhibitory markers and the subset dual-labeled with mAChRs. Volumetric stereological counting rules were implemented with inclusion/exclusion criteria to avoid counting a cell soma more than once due to the inherent errors of cell plucking and cell splitting during sectioning, as described (Schmitz and Hof, 2005). Neuronal cell bodies touching the inclusion borders (x-y top and right border of the image, and the topmost optical z slice) of the image were counted, while those touching exclusion borders were omitted. The resulting raw data counts were expressed as a density measure (neurons/mm^3^) in each cortical layer and area to yield four values per animal and marker as follows: ACC L2, ACC L3, LPFC L2, LPFC L3.

### Quantification and Colocalization of mAChR^+^ Puncta

#### Total Labeled Puncta in Neuropil

We assessed the total optical density of either muscarinic receptors (m1, m2) puncta or synaptic markers (VGAT, VGLUT1, VGLUT2) in the neuropil using the particle analysis function in FIJI/ImageJ^2^ (1997–2016); RRID:SCR_002285 (Schindelin et al., 2012). The signal threshold was determined using either the Otsu or Renyi method and was applied to all images per case. The average measure of receptor or synaptic puncta optical density was calculated for L1-L3 and expressed as percent area labeled (Schneider et al., 2012).

#### Colocalization of mAChR With Pyramidal Neuron Somatic and Dendritic Postsynaptic Compartments

We used the ROI manager and colocalization plug-in in FIJI to quantify the colocalization of mAChR^+^ on distinct somatic and dendritic compartments of labeled excitatory pyramidal neurons (MAP2, SMI-32). The segmentation editor and ROI manager in FIJI were used to isolate the somatic and dendritic compartments (ACC *n* = 10 soma/dendrite, LPFC *n* = 10 soma/dendrite) of morphologically identified MAP2^+^ pyramidal neurons in L3. The density of mAChR^+^ within these identified MAP2^+^ ROIs was performed by using particle analyses to estimate the percent area labeled within each ROI (Schneider et al., 2012).

#### Colocalization of m2^+^ Puncta on Excitatory and Inhibitory Presynaptic Terminals

We estimated the extent of pre-synaptic localization of the m2^+^ receptor on excitatory and inhibitory axon terminals by dual-labeling of m2^+^ with excitatory and inhibitory presynaptic markers: vesicular glutamatergic (VGLUT1^+^ and VGLUT2^+^) and GABAergic (VGAT^+^) transporter proteins. Additionally, in L1 and L3 of ACC and LPFC, we performed a triple-colocalization to examine the proportion of CB^+^ and PV^+^ VGAT^+^ axon terminals colocalized with m2^+^. We used the particle analysis function (Schneider et al., 2012) followed by the FIJI EZ colocalization plug-in (Stauffer et al., 2018) to calculate the degree of colocalized pixels in the neuropil using the Mander’s overlap coefficient, as previously described (Medalla and Luebke, 2015; LeBlang et al., 2020; ACC *n* = 6 cases, LPFC *n* = 6 cases). Colocalization was calculated based on Mander’s colocalization coefficients, which is the ratio of percent area colocalized over the percent area of either channel 1 or channel 2 (Stauffer et al., 2018).

#### Colocalization of m2^+^ Puncta on Compartment-Specific Inhibitory Presynaptic Terminals

In the VGAT^+^ labeled tissue, we assessed whether inhibitory inputs to specific pyramidal neurons compartments express m2^+^. We performed triple colocalization of VGAT^+^ m2^+^ with a marker for pyramidal neurons (MAP2^+^ or SMI-32^+^), using a colocalization plug-in followed by particle analyses in FIJI. We selected a random set of L3 MAP2^+^ labeled pyramidal neurons, delineated the proximal apical dendritic and somatic ROIs (as above), and ran the colocalization plug-in to create a mask of colocalized VGAT^+^ with m2^+^ puncta. Then, the percent area of these dual labeled VGAT^+^ m2^+^ puncta within each MAP2^+^ ROI was calculated using particle analyses. For SMI-32^+^ pyramidal neurons, we selected a subset with a visible somatic and dendritic label, ran the colocalization plug-in in FIJI to visualize colocalized points, and manually counted dual-labeled VGAT^+^ m2^+^ puncta along SMI-32^+^ pyramidal somata and proximal apical dendritic trunks (first 100 μm) using Neurolucida 360 software (MBF Biosciences). Puncta were identified as VGAT^+^ m2^+^ or VGAT^+^ only (ACC *n* = 10 neurons, LPFC *n* = 10 neurons). Puncta identified on the soma were normalized to the surface area while those on the dendrite were normalized to length.

We analyzed the density and length of VGAT^+^ axon “cartridges” in L2–3 of ACC and LPFC and their colocalization with m2^+^. Using Neurolucida 360 (MBF Biosciences), VGAT^+^ cartridges were identified as rows of puncta perpendicular to the pial surface (Somogyi, 1977), and counted using stereological counting procedures, and classified depending on their strength of m2^+^ expression as follows: strongly labeled m2^+^^+^ VGAT^+^ (>50% of VGAT^+^ cartridge area labeled was colocalized with m2); lightly-labeled m2^+^VGAT^+^ (≤50% of VGAT^+^ cartridge area labeled was colocalized with m2); or m2^−^ VGAT^+^. The density and proportion of each “cartridge type” were compared between areas. For each case, we exhaustively measured the length of individual VGAT^+^ cartridges in LPFC (*n* = 300 cartridges measured from six cases) and ACC (*n* = 200 cartridges from five cases). The measured lengths were averaged for each case and compared between areas. A subset of the VGAT cartridge types classified as lightly m2^+^ labeled and strongly m2^+^^+^ labeled was further measured for length and analyzed for proportion and densities (number per length of the cartridge) of individual VGAT^+^ m2^−^ and VGAT^+^ m2^+^ boutons along each cartridge.

### Statistical Analyses

All statistical analyses were conducted in MATLAB (Mathworks, Natick, MA). An outlier analysis and tests for normality (Z-score calculation and Kolmogorov-Smirnov test) were performed for each outcome measure and group. Between-area (ACC vs. LPFC) and between-layer (L1, L2, L3) comparisons of outcome measures were conducted using a Two-Way ANOVA (for layer × area and mAChR × layer within area) with a Fisher’s Least Significant Difference (LSD) *post hoc* analysis. Comparisons between somatodendritic compartments by area were performed using a multiple comparisons One-Way ANOVA with a Fisher’s Least Significant Difference (LSD) *post hoc* analysis. Between and within groups statistical comparisons of frequency distributions were performed using Chi-Squared tests and corrected for multiple comparisons using the Bonferroni method.

## Results

**Figure 1:**
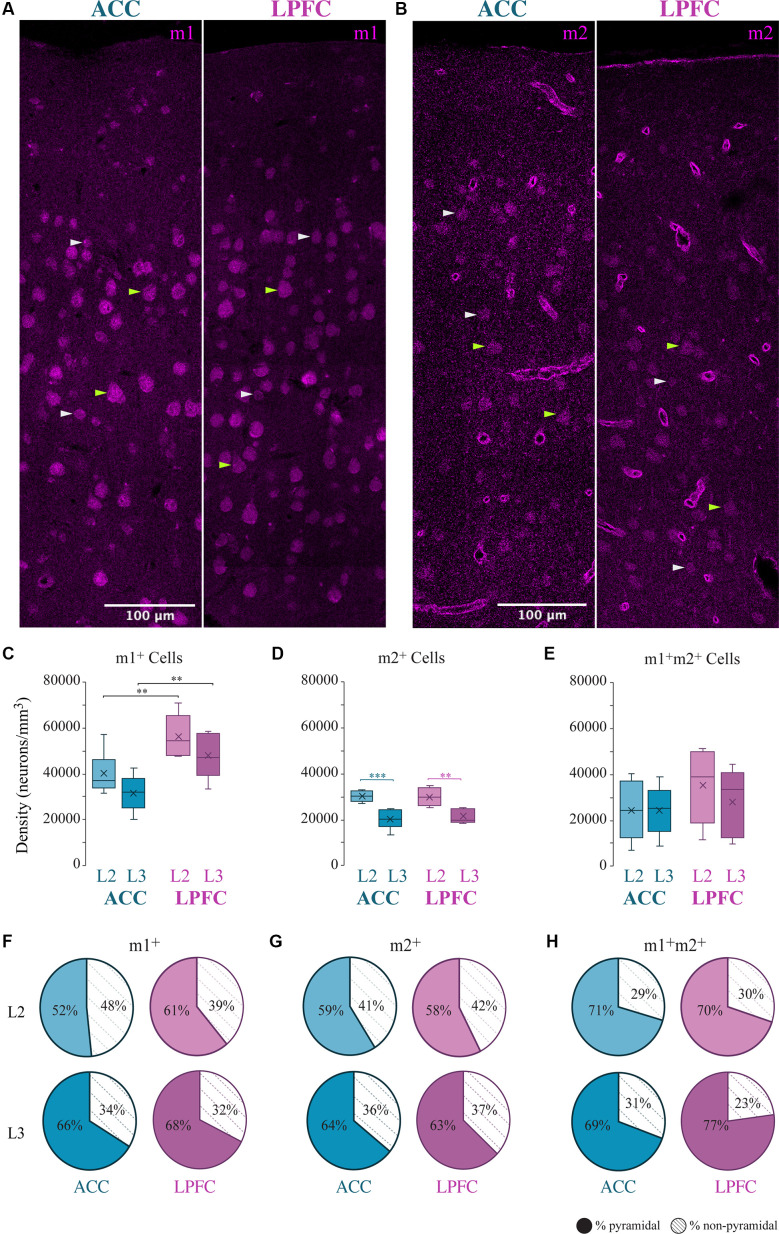
Distribution of m1^+^ and m2^+^ expressing cells in the ACC and LPFC. **(A,B)** Representative confocal image of L1-L3 showing m1^+^ **(A)** or m2^+^ **(B)** cells in ACC and LPFC. Green arrows indicate morphologically identified pyramidal neurons, white arrows are non-pyramidal cells. Scale bar = 100 μm. **(C–E)** Box and whisker density plots of m1^+^ **(C)**, m2^+^ **(D)**, or **(E)** m1^+^m2^+^ expressing cells per mm^3^ in L2 and L3 of ACC and LPFC. **(F–H)** The relative proportion of m1^+^ **(F)** or m2^+^ **(G)** or m1^+^m2^+^ **(H)** pyramidal vs. non-pyramidal cells in L2 (top) and L3 (bottom) in the ACC and LPFC were equivalent between areas and layers.**p ≤ 0.01, ***p ≤ 0.001. LPFC, lateral prefrontal cortex; ACC, anterior cingulate cortex.

We assessed the overall density distribution of the total cells expressing m1^+^, m2^+^ or both m1^+^ m2^+^ within L2 and L3 of ACC area 24 and of LPFC area 46 (Figure 1). Within the ACC, m1^+^ cells were significantly greater than cells expressing m2^+^ in L3 (*p* = 0.04, Two- Way ANOVA, *Fisher’s* LSD *post hoc*) and co-expressing m1^+^ m2^+^ in L2 (*p* = 0.01). While in LPFC L2 and L3, m1^+^ cells were significantly greater than m2^+^ (L2: *p* = 3.08 × 10^−4^, L3: *p* = 9.47 × 10^−5^) and m1^+^m2^+^ cells (L2: *p* = 2.97 × 10^−3^, L3: *p* = 1.82 × 10^−3^). Two-way ANOVA comparisons revealed significant between-area differences for m1 cells and within-area (between-layer) differences for m2^+^ cells. LPFC L2 and L3 had a significantly greater m1^+^cell density compared to ACC (LPFC vs. ACC L2: *p* = 9.37 × 10^−3^, LPFC vs. ACC L3: *p* = 8.53 × 10^−3^; Figure 1C). Within both ACC and LPFC, the density of m2^+^ cells was significantly greater in L2 compared to L3 (ACC L2 vs. L3: *p* = 1.09 × 10^−4^, LPFC L2 vs. L3: *p* = 1.43 × 10^−3^; Figure 1D). No significant differences in the density of co-expression of m1^+^ m2^+^ receptors within or between areas were found (Figure 1E). Of the total m1^+^, m2^+^, or m1^+^m2^+^ expressing cells within these areas and layers, 52–77% were identified as pyramidal, based on morphology, and the rest were non-pyramidal (Figures 1F–H).

### Distribution of m1^+^ and m2^+^ Expressing MAP2^+^ Pyramidal Neuron in ACC and LPFC

**Figure 2:**
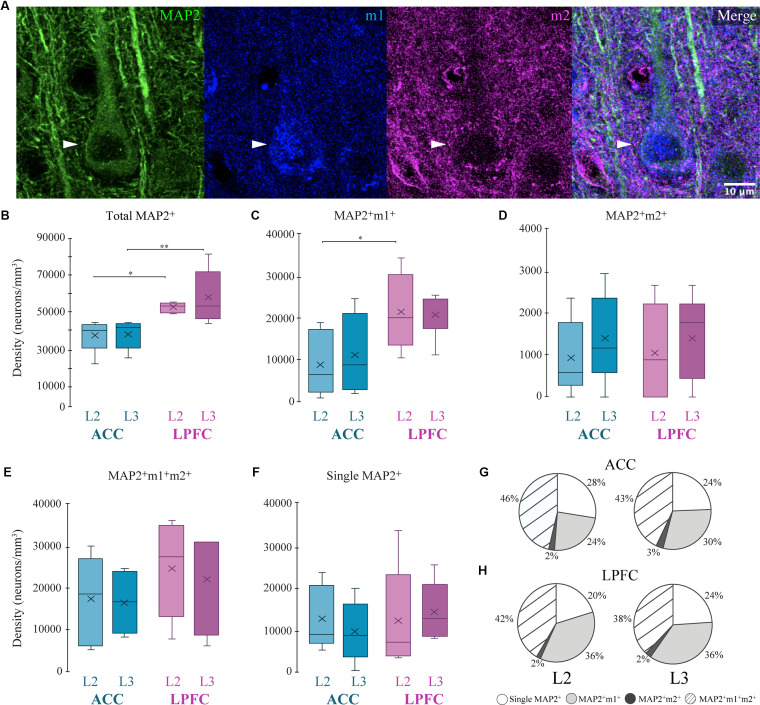
Distribution of MAP2^+^ pyramidal neurons expressing mAChR. **(A)** Representative confocal image stack illustrating the colocalization of a MAP2^+^ neuron (arrows; green) with m1 (blue) and m2 (red). Scale bar = 20 μm. **(B–F)** Box and whisker density plots of MAP2^+^ neurons within and between the ACC and LPFC are summarized as follows. **(B)** The total density of MAP2^+^ neurons was significantly greater in LPFC. **(C–E)** MAP2^+^ neurons co-labeled with either **(E)** m1^+^, **(F)** m2^+^, or **(G)** both receptors, revealed significant differences only with regard to m1^+^. **(F)** The density of single labeled MAP2^+^ neurons was equivalent within and between areas. **(G,H)** Pie charts showing the relative proportion of MAP2^+^ neurons co-labeled with either or both mAChRs. *p ≤ 0.05, **p ≤ 0.01. mAChR, muscarinic receptor.

We used the cytoskeletal protein MAP2, which strongly labels somata and dendrites of excitatory pyramidal neurons (Caceres et al., 1984; Peters and Sethares, 1991), and quantified the density of morphologically identified MAP2^+^ pyramidal neurons co-expressing either m1^+^, m2^+^, or m1^+^m2^+^ receptors (Figure 2). Within-area comparisons showed the total densities of MAP2^+^ pyramidal neurons in L2 and L3 were equivalent (Figure 2B). However, between-area comparisons showed that LPFC had a greater density of L2 and L3 MAP2^+^ pyramidal neurons compared to ACC (Two-Way ANOVA layer × area, Fisher’s LSD *post hoc*: LPFC vs. ACC L2: *p* = 0.021; LPFC vs. ACC L3: *p* = 0.003; Figure 2B). Further, in L2, LPFC had a significantly greater density of the subpopulation of MAP2^+^m1^+^ pyramidal neurons, compared to ACC (*p* = 0.018, Figure 2C). There were no significant differences in the density of other MAP2^+^ pyramidal neuron subpopulations: MAP2^+^ m2^+^ (Figure 2D), MAP2^+^ m1^+^ m2^+^ (Figure 2E), or MAP2^+^m1^−^m2^−^ neurons (Figure 2F). An assessment of the proportion of these MAP2^+^ pyramidal subpopulations (Figures 2G,H) showed that the majority of MAP2^+^ pyramidal neurons expressed m1^+^ and/or m2^+^ receptors (72–80%), and the proportions did not differ between ACC and LPFC. About 38–46% of MAP2^+^ pyramidal neurons co-expressed both m1^+^ and m2^+^ and 24–36% expressed m1^+^ alone, which was greater than the proportion of MAP2^+^ pyramidal neurons that expressed m2^+^ alone ~2% (Figures 2G,H).

### Differential Distribution of m1^+^ and m2^+^ Expressing SMI-32^+^ and CB^+^ Pyramidal Neuron Subpopulations in ACC and LPFC

We next characterized m1^+^ and m2^+^ expression on specific subclasses of pyramidal neurons expressing the neurofilament protein SMI-32^+^ as well as the calcium-binding protein, calbindin (CB^+^). SMI-32 has been shown to be strongly expressed in long–range projecting pyramidal neurons in L3 and L5, such as corticospinal neurons (Campbell and Morrison, 1989; Barbas and Garcia-Cabezas, 2016). CB, although mainly a marker for inhibitory interneurons in the monkey cortex, also lightly labels a small population of pyramidal neurons in limbic areas (Hof and Nimchinsky, 1992; DeFelipe, 1997; Kondo et al., 1999; Dombrowski et al., 2001).

**Figure 3:**
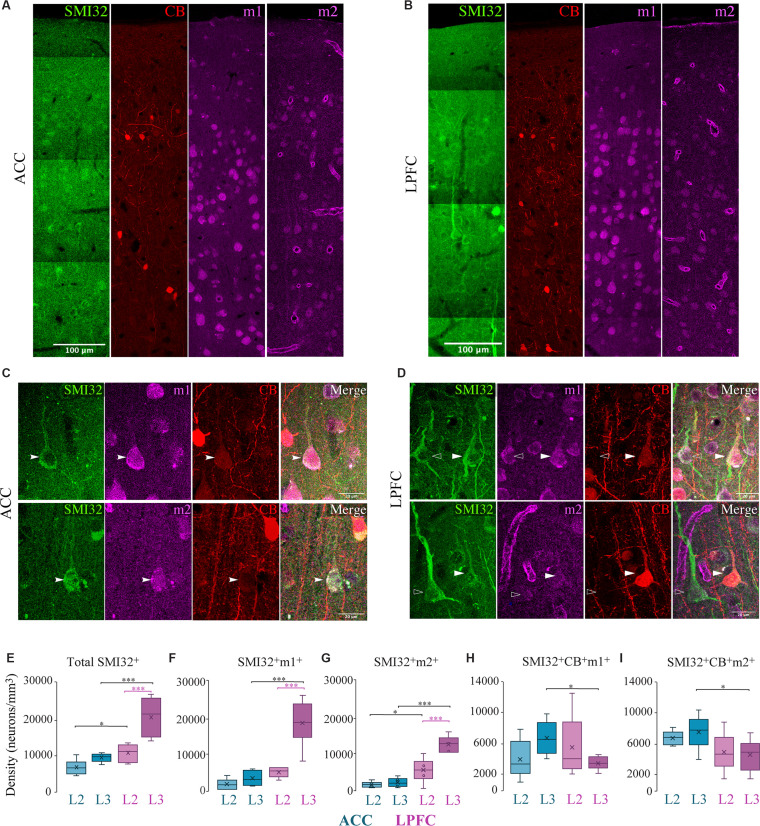
Distribution of SMI-32^+^ and CB^+^ pyramidal neuron subpopulations expressing mAChR. **(A–D)** Representative confocal images of SMI-32^+^ (green), calbindin (red), m1^+^ and m2^+^ (magenta) in layers 1–3 of **(A)** ACC and **(B)** LPFC. Scale bar: 100 μm. **(C,D)** Representative confocal images highlight the colocalization of L3 SMI-32^+^ pyramidal neuron (arrows), with either m1^+^ (*top*) or m2^+^ (*bottom*) and CB^+^ in **(C)** ACC or **(D)** LPFC. Scale bar: 20 μm. **(E–G)** Box and whisker density plots of **(E)** total SMI-32^+^ neurons, or their co-expression with either **(F)** m1^+^ or **(G)** m2^+^ revealed that the LPFC had an overall greater density compared to ACC. **(H,I)** SMI-32^+^ neurons co-expressing CB^+^ with either **(H)** m1^+^ or **(I)** m2^+^ revealed that ACC had a greater density compared to LPFC. *p ≤ 0.05, ***p ≤ 0.001.

Similar to MAP2^+^, the subpopulation of SMI-32^+^ pyramidal neurons was present in a greater density in LPFC than in ACC (Two-Way ANOVA layer × area, Fisher’s LSD *post hoc*; LPFC vs. ACC: L2 *p* = 0.04, L3 *p* = 2.59 × 10^−5^; Figures 3A–E), as shown in previous studies (Hof and Nimchinsky, 1992; Barbas and Garcia-Cabezas, 2016). In both ACC and LPFC, ~100% of the total SMI-32^+^ neurons expressed m1^+^, while 81–100% of all SMI-32^+^ neurons expressed m2^+^ (not shown). Within-area laminar comparisons showed that the density and proportion of SMI-32^+^ neurons expressing m1^+^ or m2^+^ was greater in LPFC L3 compared to L2 (LPFC L2 vs. L3: SMI32^+^m1^+^: *p* = 6.30 × 10^−10^; SMI32^+^m2^+^: *p* = 2.27 × 10^−4^, Fisher’s LSD *post hoc*; *p* = 5.03 × 10^−8^, Chi-Square and Bonferroni *post hoc*; Figures 3F,G). However, within the ACC, there was no statistical difference between the layers. Between-area comparisons showed that there was a significantly greater density of total SMI-32^+^ and SMI-32^+^m1^+^ neurons in L3 of LPFC than in ACC (LPFC vs. ACC, Fisher’s LSD *post hoc*: SMI-32^+^ *p* = 2.59 × 10^−5^, SMI-32^+^m1^+^ *p* = 2.80 × 10^−11^; Figure 3F). SMI-32^+^m2^+^ neuron densities exhibited between-area differences in both layers, with LPFC having significantly greater densities compared to ACC (LPFC vs. ACC, L2: *p* = 0.01; L3: *p* = 5.15 × 10^−7^; Figure 2G). Further, we observed a significantly greater proportion of SMI-32^+^m2^+^ in LPFC L3 compared to ACC (LPFC vs. ACC L3: *p* = 3.18 × 10^−7^, Chi-Square and Bonferroni *post hoc*).

In contrast to the predominance of SMI-32^+^m1^+^ or m2^+^ neurons in LPFC, the subset of pyramidal neurons co-expressing SMI-32^+^ and CB^+^ were present in greater density in ACC. Specifically, between-area comparison showed that SMI-32^+^CB^+^m1^+^ and SMI-32^+^CB^+^m2^+^ neurons in L3 had a significantly greater density in ACC compared to LPFC (LPFC vs. ACC L3, m1^+^: *p* = 0.02; m2^+^: *p* = 0.03, Fisher’s LSD *post hoc*; Figures 3H,I). Similarly, the proportions of SMI-32^+^CB^+^ expressing m1^+^ and m2^+^ neurons were significantly greater in L3 ACC than in LPFC: with ~65% in ACC compared to ~16% in LPFC expressing m1^+^ (*p* = 3.22 × 10^−2^, Chi-Square and Bonferroni *post hoc*), and ~74% in ACC compared to ~21% in LPFC expressing m2^+^ (*p* = 3.57 × 10^−2^; data not shown).

In summary, m1^+^ pyramidal neurons were greater in density than m2^+^ pyramidal neurons. Interestingly, the subset of SMI-32^+^ and SMI-32^+^CB^+^ pyramidal neurons expressing m1^+^/m2^+^ exhibited opposite regional distributions: SMI-32^+^m1^+^/m2^+^ neurons were greater in LPFC than ACC, while SMI-32^+^CB^+^m1^+^/m2^+^ neurons were greater in ACC than in LPFC (Figures 3E–I).

### Specificity of m1^+^ and m2^+^ Cholinergic Receptor Expression on GABAergic Interneuron Subtypes

**Figure 4:**
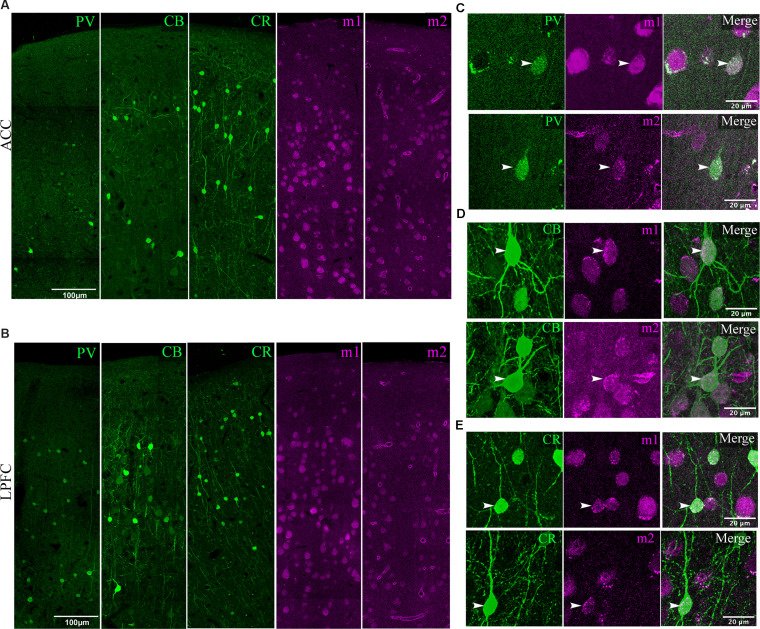
Visualization of mAChR on distinct inhibitory neurons. **(A,B)** Low magnification confocal image stacks of coronal sections in layers 1–3 of **(A)** ACC and **(B)** LPFC showing the distribution of inhibitory subtypes (green) PV^+^, CB^+^, CR^+^, and muscarinic receptors (magenta) m1^+^ and m2^+^. Scale bar: 100 μm. **(C–E)** Representative high magnification confocal images of inhibitory neurons (arrows) labeled **(C)** PV^+^, **(D)** CB^+^, and **(E)** CR^+^ that co-express either m1^+^ (*top*) or m2^+^ (*bottom*) receptors. Scale bar: 20 μm.

**Figure 5:**
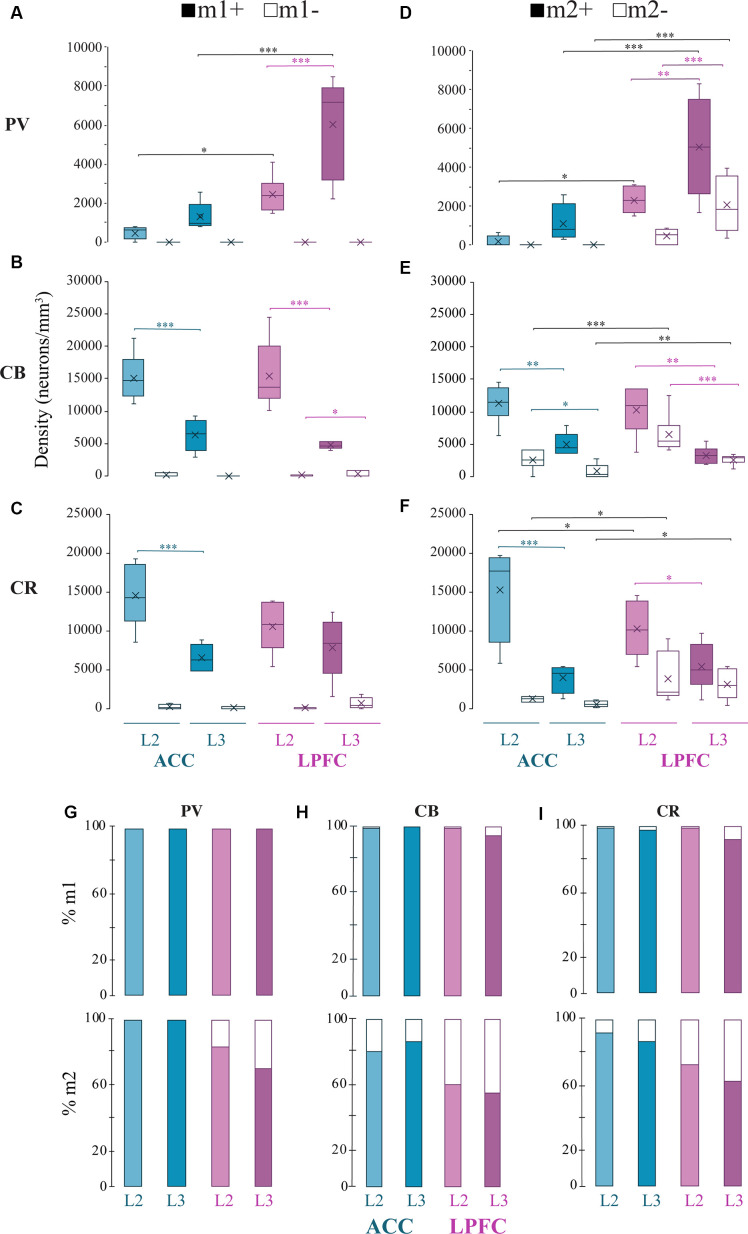
Distribution of mAChR expressing inhibitory neuron subtypes. **(A–C)** Box and whisker cell density plots of interneuron subtypes with or without m1^+^ where **(A)** m1^+^ PV^+^ was greater in L3 vs. L2, while **(B,C)** showed a greater density of m1^+^CB^+^ and m1^+^ CR^+^ in L2 vs. L3 in the ACC and the LPFC. Similarly, **(D–F)** showcase cell density plots of interneuron subtypes with or without m2^+^ where the density of **(D)** m2^+^PV^+^ was greater in L3 vs. L2 of the LPFC. **(E)** m1^+^CB^+^ and m2^+^CB^+^ neurons were denser in L2 vs. L3 in both areas. Lastly, **(F)** m2^+^CR^+^ interneurons were denser in L2 vs. L3 in ACC, while both m2^+^CR^+^ and m2^+^CR^+^ neurons showed greater densities in L2 vs. L3 in LPFC. **(G–I)** Among the three interneuron classes **(G)** PV^+^, **(H)** CB^+^, and **(I)** CR^+^, the relative proportion of m1^+^ (*top)* was similar across areas and interneuron subtypes while m2^+^ (*bottom)* was overall greater in ACC compared to LPFC. *p ≤ 0.05, **p ≤ 0.01, ***p ≤ 0.001.

Previous studies have shown that different neurochemical classes of inhibitory neurons in rhesus monkeys expressing the calcium-binding proteins calbindin (CB^+^), parvalbumin (PV^+^), and calretinin (CR^+^) differ in density and laminar distribution across prefrontal cortices (Dombrowski et al., 2001). Here, we assessed the density of the specific interneuron subpopulations that expressed m1 or m2 mAChR subtypes (Figures 4, 5).

Consistent with previous work on the total density of PV^+^ interneurons (Dombrowski et al., 2001), we observed that PV^+^m1^+^ and PV^+^m2^+^ subpopulations showed a significantly greater density in LPFC compared to ACC (m1^+^ L2: *p* = 0.04, L3: *p* = 8.3 × 10^−6^, m2^+^L2 *p* = 0.03; *p* = 2.7 × 10^−4^; Two-Way ANOVA for each area and marker, Fisher’s LSD *post hoc*; Figures 4; 5A,D). Further, within LPFC, a laminar difference was observed, with the density of both PV^+^m1^+^ (*p* = 2.02 × 10^−4^, Figure 5A) and PV^+^m2^+^ (*p* = 3.42 × 10^−3^, Figure 5D) interneurons being greater in L3 compared to L2.

For the non-pyramidal CB^+^ interneurons, the density of m1^+^ and m2^+^ expressing subpopulations significantly differed between layers, but not between areas (Figures 4, 5B,E). Between-layer comparisons within each area showed that L2 had a significantly greater density of CB^+^ m1^+^ and CB^+^ m2^+^ interneurons compared to L3 (ACC: m1^+^ *p* = 1.13 × 10^−5^, m2^+^ *p* = 8.89 × 10^−3^; LPFC: m1^+^ *p* = 1.24 × 10^−4^, m2^+^ *p* = 6.68 × 10^−3^; Figures 5B,E). Furthermore, within ACC L2, a greater density of CB^+^ interneurons colocalized with m1^+^ than m2^+^ (CB^+^m1^+^ vs. CB^+^m2^+^: *p* = 0.007; Figures 5B,E).

The density of m1^+^ and m2^+^ expressing CR^+^ interneurons showed significant between-layer and between-area differences (Figures 4, 5C,F). Within the ACC, the density of CR^+^m1^+^ (*p* = 9.09 × 10^−4^) and CR^+^m2^+^ (*p* = 9.86 × 10^−5^) interneurons was significantly greater in L2 compared to L3 (Figures 5C,F). However, the LPFC had a significant laminar difference only for CR^+^m2^+^ interneurons (L2 vs. L3 *p* = 0.03; Figure 5F). Between-area comparisons showed that in L2, ACC had a greater mean density of CR^+^ m2^+^ interneurons compared to the LPFC (*p* = 0.04, Figure 5F).

We compared the relative proportion of m1^+^ and m2^+^ expressing interneurons within each interneuron subtype. Similar to pyramidal neurons, the majority of inhibitory neurons expressed mAChRs, with the proportion expressing m1^+^ greater than those expressing m2^+^ (Figures 5G–I). The proportion of m1^+^ expressing subpopulations did not differ across neurochemical interneuron types or between cortical areas (100% of all PV; 95–100% of all CB and 92–99% of all CR). However, the ACC and LPFC differed in the proportion of m2^+^ expressing subpopulations depending on the interneuron subclass and layer. In the ACC, 100% of PV^+^ interneurons express m2^+^ (presumably colocalized with m1^+^) while in the LPFC, 84% of PV^+^ interneurons in L2 and 71% in L3 expressed m2^+^, in proportions significantly less than ACC (L2: *p* = 1.59 × 10^−5^, L3: *p* = 3.45 × 10^−10^, Fisher Exact Test; Figure 5G). Similar to PV, between-area comparison revealed a significantly higher proportion of CB^+^m2^+^ interneurons in L2 (81% in ACC; 61% in LPFC) and L3 (87% in ACC; 56% in LPFC) of ACC than in LPFC (L2 *p* = 1.67 × 10^−3^, L3 *p* = 8.17 × 10^−7^, Fisher’s LSD *post hoc*; Figure 5H). A significantly higher proportion of CR^+^m2^+^ interneurons was also found in ACC than in LPFC (L2: 3.77 × 10^−3^, L3: 1.1 × 10^−5^). In the ACC, the proportion of CR^+^m2^+^ accounted for 92% of L2 and 87% L3 of the total CR^+^ interneurons (Figure 5I), similar to the proportion found in m1^+^ expressing CR^+^ interneurons. In the LPFC, CR^+^m2^+^ interneurons (73% of all CR^+^ in L2 and 63% in L3) represented a lower proportion of the total CR^+^ population than the CR^+^ m1^+^ interneurons (99% of all CR^+^ in L2, 88% in L3; Figure 5I).

### Subcellular Localization of mAChR Along MAP2^+^ Pyramidal Neurons

**Figure 6:**
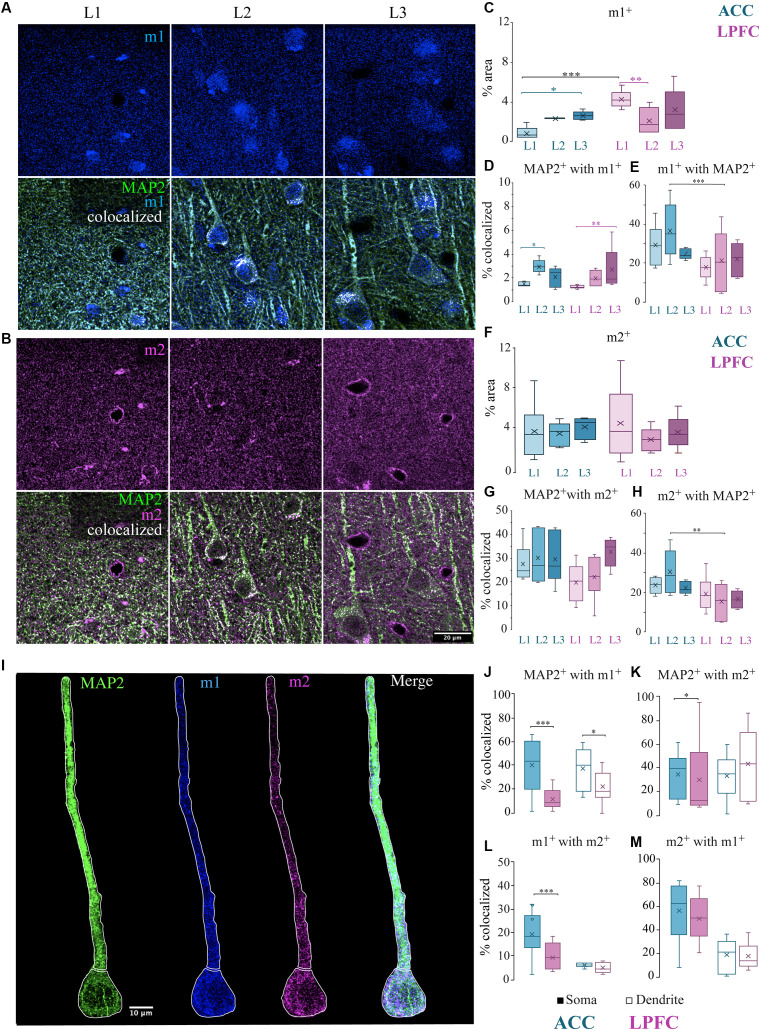
Localization of mAChR on MAP2^+^ pyramidal neurons. **(A,B)** Representative confocal images of the distribution in the L1-L3 neuropil of either **(A)** m1^+^ receptors (blue) *(top)* and MAP2^+^ (green) m1^+^ (blue) with colocalized pixels (white) *(bottom)* or **(B)** m2^+^ receptors (magenta) *(top)* and MAP2^+^ (green) m2^+^ (magenta) with colocalized pixels (white) *(bottom).* Scale bar: 20 μm. **(C)** Box and whisker plots showing density (% area) of m1^+^ label and **(D)** colocalization coefficients representing % of MAP2^+^ with m1^+^ and **(E)** % of m1^+^ with MAP2. In ACC, L2 had a significantly greater percent of MAP2^+^ with m1^+^ compared to L1 while LPFC had a significantly greater percent in L3 compared to L1 **(D)**. ACC L2 had a significantly greater percent of m1^+^ colocalized with MAP2^+^ than LPFC **(E)**. **(F)** Box and whisker plots showing density (% area) of m2^+^ label and **(G)** colocalization coefficients representing % of MAP2^+^ with m2^+^ and **(H)** % of m2^+^ with MAP2. ACC L2 had a significantly greater percent of m2^+^ colocalized with MAP2^+^ than LPFC **(H)**. **(I)** Representative confocal images showing proximal apical dendritic and somatic ROIs illustrated by white outline of representative MAP2^+^ neuron (green) co-labeled with m1^+^ (blue) and m2^+^ (magenta). Scale bar: 10 μm. **(K–M)** Box and whisker plots of percent area of m1 and m2 in the somatic (filled) or dendritic (unfilled) ROIs of individual cells. **(J–K)** The colocalized % of MAP2^+^ with either **(J)** m1^+^ or **(K)** m2^+^ was significantly greater in the ACC somatic compartment, but only **(J)** MAP2^+^ m1^+^ was greater in the dendritic compartment of the ACC compared to the LPFC. **(L,M)** The colocalized % of **(L)** m1^+^ with m2^+^ or **(M)** m2^+^ with m1^+^. *p ≤ 0.05, **p ≤ 0.01, ***p ≤ 0.001.

Cholinergic modulation of cortical circuits is dependent on the location of mAChRs on different morphological compartments of neurons outside of the soma (Mrzljak et al., 1993; Disney et al., 2006). Thus, we examined the optical density of m1^+^or m2^+^ receptor expression within the neuropil (containing neurites and synapses) of L1-L3 in both LPFC and ACC, quantified as the percent area labeled within total tissue volume examined (Figures 6A–C,F). Within area, the percent area of m1^+^ was significantly greater in ACC L3 than in L1 (*p* = 0.03, Two-Way ANOVA area x layer, Fisher’s LSD *post hoc*), but in LPFC was significantly greater in L1 than in L2 (*p* = 7.5 × 10^−3 ^; Figure 6C). Further, a between-area comparison revealed a significantly greater density of m1^+^ in L1 of the LPFC compared to ACC (*p* = 1.7 × 10^−4^; Figure 6C). The percent area labeled with m2^+^ was equivalent across areas and layers of ACC and LPFC (Figure 6F).

We then examined the percent area of colocalization of MAP2^+^ with either m1^+^ or m2^+^ within the total tissue volume in L1, L2, and L3 of ACC and LPFC (Figures 6A,B). Within-area comparisons found a significantly greater percent area of MAP2^+^ colocalized with m1^+^ in ACC L2 than in L1 (*p* = 0.02, main effect layer, Fisher LSD *post hoc*) and in LPFC L3 than in L1 (*p* = 9.41 × 10^−3^; Figure 6D); however, there were no between-area differences. There were no significant within- or between-area differences in the percent area of MAP2^+^ colocalized with m2^+^ (Figure 6G). However, among the total m1^+^ or m2^+^ label in the neuropil, a greater percent was colocalized with MAP2^+^ in ACC L2 compared to LPFC (Figures 6E,H). These data suggest that among all structures bearing m1^+^ and m2^+^ receptors in L2, MAP2^+^ dendrites represent a larger proportion of the potential ACh modulatory target in ACC than in LPFC.

The data in the neuropil suggested differential subcellular localization of m1 and m2 receptors within ACC and LPFC. Thus, we further investigated the specific localization and density of m1^+^ and m2^+^ on distinct subcellular compartments of individual neurons. We performed a segmentation of the proximal apical dendrite and soma of individual L3 MAP2^+^ pyramidal neurons (Figure 6I). We then quantified the density (% area) of colocalized label within segmented MAP2^+^ subcellular ROIs and assessed overlap of label as follows: (1) MAP2^+^m2^+^, (2) MAP2^+^m1^+^, and (3) MAP2^+^m1^+^m2^+^ in the ACC (*n* = 10 soma/dendrite) and LPFC (*n* = 10 soma/dendrite). Compared to the LPFC, the ACC had a greater density of m1^+^ label within MAP2^+^ somatic and dendritic ROIs (somatic: *p* = 8.87 × 10^−6^, dendritic: *p* = 1.52 × 10^−3^; One-Way ANOVA; Figure 6J), while m2^+^ within MAP2^+^ was only significant in the somatic ROI (*p* = 2.31 × 10^−4^; One-Way ANOVA; Figure 6K). Within-area comparisons between the two compartments (soma vs. dendrite) showed an equivalent m2^+^/m1^+^ and m1^+^/m2^+^ colocalization (Figures 6L,M). However, between-area comparisons showed that post-synaptic m1^+^ receptor had a significantly higher percentage of colocalization with presynaptic m2^+^ specifically in the somatic compartment of ACC MAP2^+^ pyramidal neurons compared to LPFC (*p* = 4.49 × 10^−4^) (Figure 6L). Although we found that there was a greater cell density of MAP2^+^ pyramidal neurons in the LPFC (Figure 2D), the m1^+^ and m2^+^ receptor expression density *per individual* MAP2^+^ neuron was greater in ACC than LPFC.

### Presynaptic Location of m2^+^ on Excitatory and Inhibitory Axon Terminals

**Figure 7:**
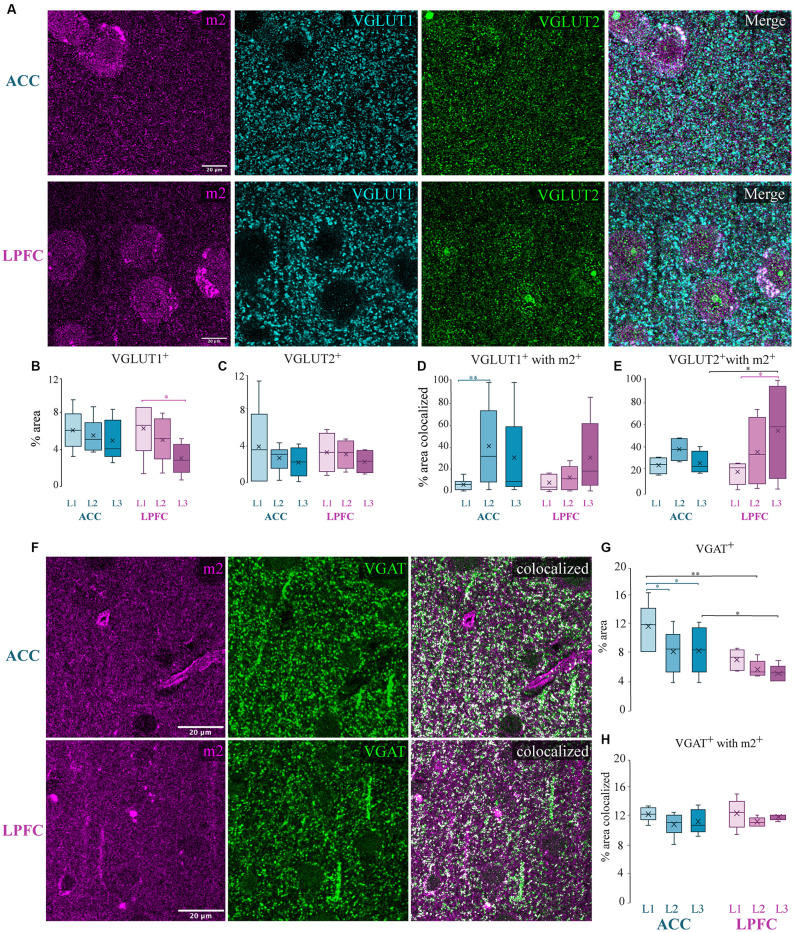
Presynaptic m2^+^ colocalization with excitatory and inhibitory axon terminals. **(A)** Representative confocal images of m2^+^ (magenta), VGLUT1^+^ (cyan), and VGLUT2^+^ (green) in the ACC (top) and LPFC (bottom). **(B,C)** The density (% area labeled) of VGLUT1^+^ puncta **(B)** was significantly greater in L1 of LPFC compared to L3 while VGLUT2^+^ **(C)** boutons were equivalent across layers and areas. **(D,E)** Colocalization coefficient (% area colocalized) of VGLUT1^+^ and VGLUT2^+^ with m2^+^. **(D)** In ACC, a significantly greater percent of VGLUT1^+^ boutons expressing m2^+^ was found in L2 compared to L1. **(E)** In LPFC, a significantly greater percent of VGLUT2^+^ boutons expressing m2^+^ was found in L3 compared to L1. **(F)** Representative confocal images of m2^+^ (magenta) and VGAT^+^ (green), and colocalized particles in white. Scale bar 20 μm. **(G)** % area label showing that the laminar density of VGAT^+^ was significantly greater within the ACC. **(H)** Colocalization coefficient showing % area of VGAT^+^ colocalized with m2^+^: approximately 11% of inhibitory terminals were colocalized with m2^+^ in both the ACC and LPFC. *p ≤ 0.05, **p ≤ 0.01.

Although a small subset of m1 receptors can also be located presynaptically, evidence has shown that most of the presynaptic action of ACh is mediated by m2 receptors (Sarter et al., 2009; Colangelo et al., 2019). The m2^+^ muscarinic subtype is the predominant receptor found on presynaptic glutamatergic and GABAergic axon terminals (Sarter et al., 2009; Colangelo et al., 2019), where they act to suppress neurotransmitter release (Mrzljak et al., 1993; Salgado et al., 2007). We thus examined the colocalization of m2^+^, with markers for excitatory and inhibitory axon terminals in the cortex (Mrzljak et al., 1993; Salgado et al., 2007). We assessed the optical density and m2^+^ colocalization of excitatory vesicular glutamatergic transporters 1 (VGLUT1) and 2 (VGLUT2), which label axon terminals from putative cortical and subcortical structures, respectively (Fremeau et al., 2004; Hur and Zaborszky, 2005; Hackett et al., 2011; Timbie and Barbas, 2015; Figure 7A). We found a significantly greater density (% area) of VGLUT1^+^ terminals in L1 compared to L3 of the LPFC (*p* = 0.021, Two-Way ANOVA area × layer, Fisher’s LSD *post hoc*; Figure 7B), while the density of VGLUT2^+^ terminals was equivalent across layers and areas (Figure 7C). Our data showed that within the supragranular layers, 18–37% of VGLUT1^+^ and VGLUT2^+^ excitatory terminals were colocalized with m2^+^ (Figures 7D,E). A significantly greater percent colocalization of VGLUT1^+^ boutons with m2^+^ in L2 compared to L1 was found within ACC (L1 vs. L2: *p* = 0.012; Two-Way ANOVA, Fisher LSD *post hoc*; Figure 7D), while VGLUT2^+^ colocalized with m2^+^ in LPFC L3 was greater compared to L1 (L1 vs. L3 LPFC: *p* = 0.015; Figure 7E). Between-area differences were found for the percent of VGLUT2^+^ boutons colocalized with m2^+^. Specifically, VGLUT2^+^ colocalized with m2^+^ in LPFC L3 was greater compared to ACC (L3 ACC vs. LPFC: *p* = 0.017; Figure 7E).

We next quantified the colocalization of m2 with vesicular GABA transporter (VGAT), a selectively expressed protein in GABAergic axon terminals (Chaudhry et al., 1998; Figure 7F). Consistent with our previous data (Medalla et al., 2017), we found that ACC had a significantly greater density of VGAT^+^ puncta compared to LPFC (*p* = 0.02; Figure 7G). Further, within ACC, the density of VGAT^+^ puncta was greater in L1 compared to L2 and L3 (L1 vs. L2: *p* = 4.9 × 10^−4^ and L1 vs. L3: *p* = 3.4 × 10^−4^; Figure 7G). Colocalization analyses of the percent area of total VGAT^+^ colocalized with m2^+^ in the neuropil revealed no differences between areas and layers, with m2^+^ receptor localization on approximately 11% of inhibitory terminals in the supragranular layers (Figure 7H).

### Differential m2^+^ Colocalization With Lamina-Specific CB^+^ and PV^+^ Inhibitory Terminals

**Figure 8:**
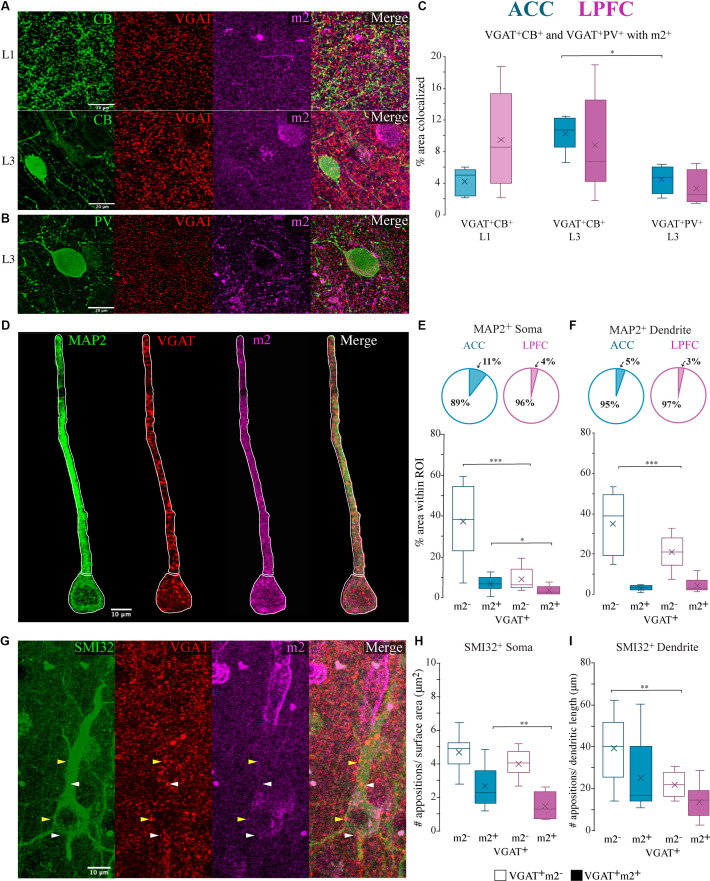
Co-localization of m2^+^ with neurochemically distinct and compartment-specific inhibitory axon terminals. **(A,B)** Representative confocal images of VGAT^+^ (red), m2^+^ (magenta) terminals with terminals from either **(A)** CB (green) in L1 *(top)* or L3 *(bottom)* or **(B)** PV (green) in L3. **(C)** Box and whisker plots of % area of m2 colocalization with VGAT^+^/CB^+^ in L1 (light hue) and L3 (dark hue) and with VGAT^+^/PV^+^ L3 (dark hue). Within the ACC, L3 had significantly greater colocalization of CB^+^ VGAT^+^ terminals with m2^+^ than PV^+^ VGAT^+^ with m2^+^. **(D–F)** VGAT + /m2 + terminals apposed (putative synapses) to specific somatodendritic MAP2^+^ compartments. **(D)** Representative confocal image of L3 MAP2^+^ neuron (green), VGAT^+^ (red), m2^+^ (magenta). White outline illustrates the ROIs of the proximal apical dendrite and the soma. Scale bar 10 μm. **(E,F)** The relative proportion *(top)* and density* (bottom)* of colocalized VGAT^+^m2^−^ and VGAT^+^m2^+^ appositions on the **(E)** soma and **(F)** proximal apical dendrite of MAP2^+^ neurons in the ACC (*n* = 10 cells) and LPFC (*n* = 10 cells). **(G)** Representative confocal image of L3 SMI-32^+^ neuron (green), VGAT^+^ (red), m2^+^ (magenta) showing proximal dendritic and somatic VGAT^+^ appositions. White arrows denote examples of VGAT^+^ only appositions and yellow arrows denote VGAT^+^m2^+^ apposition. Scale bar 20 μm. **(H,I)** The density of VGAT^+^ m2^+^ appositions (putative synapses) per cell was significantly greater in the **(H)** somatic (appositions/μm^2^) but not in the **(I)** dendritic (appositions/ μm) compartments of neurons in the ACC compared to the LPFC. *p ≤ 0.05, **p ≤ 0.01, ***p ≤ 0.001.

Inhibitory synapses on specific compartments of pyramidal neurons are conferred by neurochemically-distinct interneurons expressing CB^+^ and PV^+^ (DeFelipe, 1997; Kubota et al., 2016). We assessed the proportion of CB^+^ and PV^+^ VGAT^+^ axon terminals colocalized with m2^+^ in L1 and L3 (Figures 8A–C) using triple EZ-colocalization (Stauffer et al., 2018). Within the supragranular layers, L1 contains mainly distal apical dendrites of pyramidal neurons, while L3, is a more heterogeneous population of dendrites with include proximal dendritic segments and somata (Spruston, 2008). Given that CB interneurons primarily target the distal dendrites (Kawaguchi and Kubota, 1998; DeFelipe et al., 1999) we assessed the percent VGAT^+^ CB^+^ puncta with m2^+^ in L1 and L3. Given that PV axons densely target the proximal/perisomatic regions of neurons in L3 and are sparse in L1 (Kawaguchi and Kubota, 1998; Freund and Katona, 2007; Bartos and Elgueta, 2012) we assessed the colocalization of VGAT^+^PV^+^ puncta with m2^+^ in L3. The proportion of CB^+^ and PV^+^VGAT^+^ inhibitory terminals expressing m2^+^ showed no significant between-area differences (Figure 8C). Within-area, between-subtype comparisons showed that in L3 of ACC there was a greater percentage of VGAT^+^CB^+^ with m2^+^ compared to VGAT^+^ PV^+^ with m2^+^ (*p* = 0.043, Two-Way ANOVA, Fisher’s LSD *post hoc*; Figure 8C).

### Colocalization of m2^+^ With GABAergic Terminals Onto Pyramidal Neuron Subcellular Compartments

We quantified the density of VGAT^+^ m2^−^ and VGAT^+^ m2^+^ appositions on proximal apical dendritic or somatic ROI compartments of individual L3 MAP2^+^ (Figure 8D) and SMI-32^+^ pyramidal neurons (Figure 8G) in the ACC (*n* = 10 MAP2^+^ cells, *n* = 10 SMI-32^+^, from five cases) and LPFC (*n* = 10 MAP2^+^ cells, *n* = 10 SMI-32^+^ cells, from five cases). Compared to LPFC, we found that ACC L3 MAP2^+^ neurons had a significantly higher density of VGAT^+^m2^−^ puncta on the soma and apical dendrite (ACC vs. LPFC: soma: *p* = 8.60 × 10^−6^, apical dendrite: *p* = 8.60 × 10^−6^, One-Way ANOVA; Figures 8E,F). However, for VGAT^+^m2^+^ appositions, the two areas were equivalent with regards to appositions on proximal apical dendrites (Figure 8F), but differed with regards to appositions on the soma. The ACC had a higher density (% area) of double-labeled VGAT^+^ m2^+^ puncta as apposed to the somatic compartment (ACC vs. LPFC soma *p* = 0.03; Figure 8E). The population of VGAT^+^ m2^+^ in the somatic compartment represents ~11% of the total VGAT^+^ appositions in ACC neurons, which is significantly greater than LPFC neurons, with only ~4% of the total VGAT^+^ appositions co-expressing m2^+^ (Figure 8E).

Consistent with patterns seen with MAP2^+^ pyramidal neurons, the subpopulation of L3 SMI-32^+^ pyramidal neurons had greater densities of VGAT^+^ m2^−^ appositions on proximal apical dendrites (ACC vs. LPFC: *p* = 9.82 × 10^−3^; Figure 8I) and VGAT^+^m2^+^ perisomatic appositions in ACC compared to LPFC (ACC vs. LPFC soma: *p* = 0.01; Figure 8H). In summary, these data show that m2^−^ proximal dendrite/perisomatic inhibitory inputs and the subset of m2^+^ perisomatic inhibitory inputs are greater in ACC than LPFC.

### m2^+^ Localization on Inhibitory VGAT^+^ Axon Cartridges

**Figure 9:**
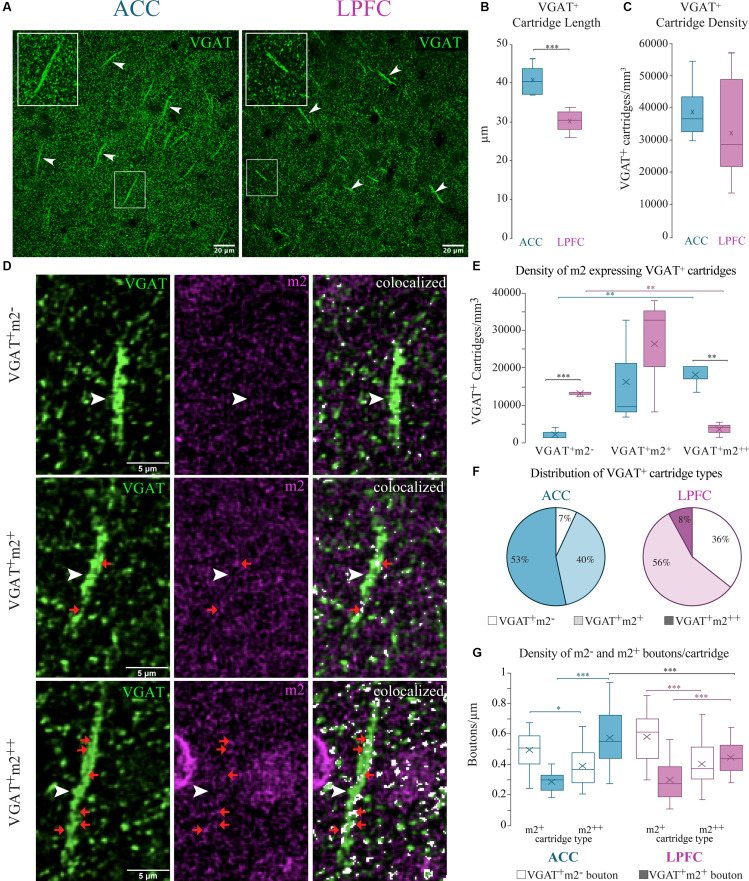
Differential m2^+^ expression of VGAT^+^ cartridges in the ACC and LPFC. **(A)** Representative confocal image of distinct VGAT^+^ cartridges (arrow) in ACC (*left*) and LPFC (*right*). Boxed insets are shown in higher magnification (in the upper left corner of each image) to highlight the difference in length of VGAT^+^ cartridges between the two areas. **(B)** The ACC revealed a significantly greater cartridge length compared to the LPFC. **(C)** The density of VGAT^+^ cartridges (number per mm^3^) was equivalent between the two areas. **(D)** Representative image of either VGAT^+^ only (*top)*, VGAT^+^ with lightly-labeled m2^+^ (*middle*) or with strongly-labeled m2^+^^+^ (*bottom*), the white arrows show the VGAT^+^ cartridges, and examples of VGAT^+^m2^+^ boutons along each cartridge are indicated by red arrows. Note that m2^+^ and m2^+^^+^ cartridges are made up of rows of both VGAT^+^m2^−^ and VGAT^+^m2^+^ boutons distributed along the length of each cartridge. Scale bar 5 μm. **(E)** The density of VGAT^+^ only cartridges was greater in the LPFC, while the ACC had greater density of m2^+^^+^ VGAT cartridges. **(F)** The relative proportion of VGAT^+^ only cartridges (*white*), lightly-labeled m2^+^ (*light colored*) or strongly-labeled m2^+^^+^ (*dark colored*) VGAT^+^ cartridges in ACC (*left)* and LPFC (*right).* *p≤ 0.05. **(G)** The density of individual VGAT^+^m2^−^ (unfilled bars) and VGAT^+^m2^+^ (filled bars) boutons along the length of distinct VGAT^+^ cartridge types classified as either lightly-labeled m2^+^ or strongly-labeled m2^+^^+^ in ACC and LPFC. *p ≤ 0.05, **p ≤ 0.01, ***p ≤ 0.001.

Axo-axonal GABAergic inputs to the axon initial segment confer a functionally powerful mode of inhibition (Somogyi, 1977; DeFelipe et al., 1989; Inan et al., 2013). These specialized inhibitory synapses can be identified by the presence of VGAT^+^ axon “cartridges”—puncta arranged in rows perpendicular to the pial surface (Somogyi, 1977) that are about 26–46 μm in length (Figure 9A). We found that the mean length of VGAT^+^ cartridges was significantly longer in the ACC (*n* = 200 cartridges from 5 cases) compared to the LPFC (*n* = 300 cartridges from 6 cases; *p* = 7.6 × 10^−5^, One-Way ANOVA; Figure 9B). However, there was no between-area difference in the density of VGAT^+^ cartridges in the neuropil quantified using stereological counting procedures (Figure 9C).

Using stereological counting procedures, we quantified and classified L3 VGAT^+^ cartridges depending on their strength of expression of m2^+^ as follows: strongly labeled m2^+^^+^ (>50% of VGAT^+^ cartridge area labeled was colocalized with m2); lightly-labeled m2^+^ (≤50%); or m2^−^/VGAT^+^ only (Figure 9D). Among the total number of VGAT^+^ cartridges in the ACC, the majority were m2^+^^+^ (53%) or m2^+^ cartridges (40%) and the minority did not express m2^−^ (VGAT^+^ only, 7%; *p* = 4.7 × 10^−8^, Chi-Square, and Bonferroni *post hoc*, Figure 9F). This was in marked contrast to the LPFC where the majority of VGAT^+^ cartridges were either lightly-labeled with m2^+^ (56%) or did not express m2^−^ (VGAT^+^ only, ~36%), and the m2^+^^+^ cartridges represented the minority (8%; *p* = 2.7 × 10^−8^). Between-area comparisons revealed that the ACC had a significantly greater density (*p* = 4.8 × 10^−3^, One-Way ANOVA) and proportion (*p* = 2.10 × 10^−12^, Fisher Exact Test) of m2^+^^+^ cartridges compared to LPFC, while the LPFC had greater density (*p* = 4.27 × 10^−4^, One-Way ANOVA) and proportion (*p* = 6.50 × 10^−7^, Fisher Exact Test) of m2^−^ /VGAT^+^ only cartridges (Figures 9E,F). We assessed whether the VGAT^+^ cartridges classified as lightly m2^+^ labeled vs. strongly m2^+^^+^ labeled in the two areas differed in their densities of individual VGAT^+^m2^−^ and VGAT^+^m2^+^ boutons per cartridge. VGAT^+^m2^−^ and VGAT^+^m2^+^ boutons were distributed randomly along the length of each cartridge. Within both areas, cartridges classified as strongly expressing m2^+^^+^ had a significantly greater proportion (data not shown; ACC: *p* = 2.23 × 10^−8^; LPFC: *p* = 8.23 × 10^−10^) and density of VGAT^+^m2^+^ labelled boutons compared to the lightly labelled m2^+^ cartridge type (ACC: *p* = 8.21 × 10^−9^; LPFC: *p* = 3.26 × 10^−5^, Two-Way ANOVA, Fisher LSD *post hoc*; Figure 9G). About ~52–59% of VGAT^+^ boutons in each m2^+^^+^ cartridge, but only ~33–37% in each m2^+^ cartridge, expressed m2. Significant between-area differences were found only for m2^+^^+^ cartridges, with the density of VGAT^+^m2^+^ labelled boutons greater in ACC than in LPFC m2^+^^+^ cartridges (Two-way ANOVA, “area” x “cartridge type” interaction, *p* = 0.03; m2^+^^+^ cartridges in ACC vs. LPFC *p* = 0.0006, Fisher LSD *post hoc*; Figure 9G). Overall, these data show that the densities of L3 VGAT^+^ cartridges strongly expressing m2^+^^+^ receptors in the neuropil and of VGAT^+^m2^+^ labeled boutons within these cartridges were greater in ACC than in the LPFC.

### Discussion

**Figure 10:**
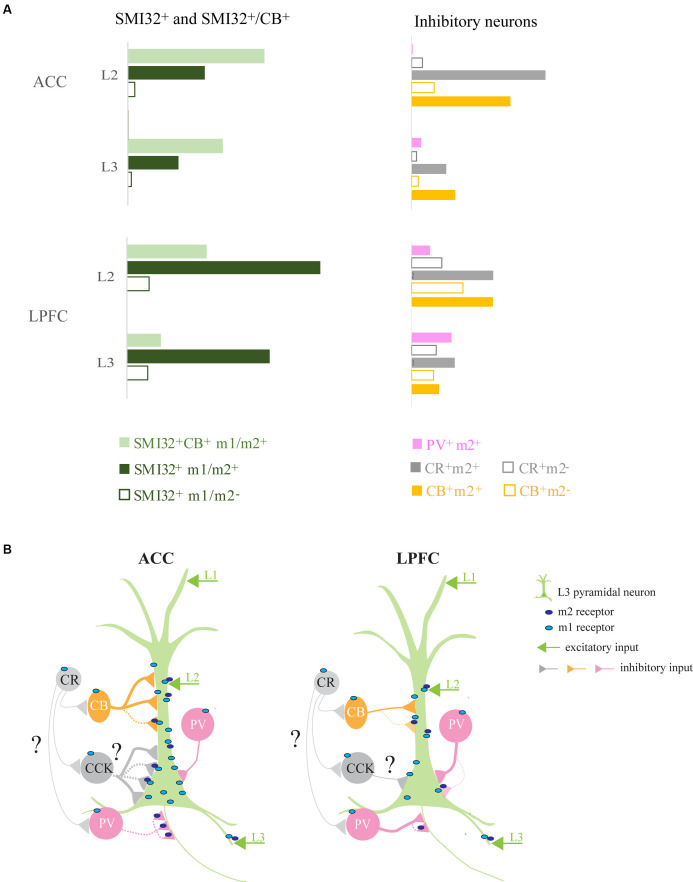
Summary of ACC and LPFC micro-circuitry influenced by muscarinic receptors. **(A)** Normalized densities of differentially distributed excitatory and inhibitory neuronal populations expressing mAChRs in ACC and LPFC. LPFC had a greater density of m1^+^/m2^+^ SMI-32^+^ while ACC had a greater density of m1^+^/m2^+^ SMI-32^+^ CB^+^ pyramidal neurons. The two areas differed with regards to m2^+^ inhibitory neurons, with inhibitory neurons in the ACC having a greater extent of m2^+^ expression than in the LPFC. **(B)** Micro-circuit schematic based on the main findings of the subcellular distribution of postsynaptic m1^+^ on MAP2^+^ dendrites and m2^+^ inhibitory terminals on specific somatodendritic and axonal compartments. The number of connections and line thickness represents the relative strength of connection, while the dotted line indicates m2 mediated pre-synaptic suppression. Compared to LPFC, L3 ACC pyramidal neurons had a greater density of m1^+^ on the dendritic and somatic compartments. The dendrites of these ACC pyramidal neurons had a greater density of total VGAT^+^, including m2^+^VGAT^+^, inhibitory inputs likely from CB^+^ (yellow) neurons. The LPFC pyramidal neurons received a lower density of perisomatic input, mostly belonging to PV^+^ interneurons (Medalla et al., 2017), and only a small subset expressed m2^+^ (pink). In contrast, the ACC had a greater density of VGAT^+^m2^+^ and VGAT^+^m2^−^ inhibitory inputs, presumably from non-PV basket cells (great) with a subset likely from CCK^+^ interneurons (Medalla et al., 2017). Note that the m2 localization on CR and CCK terminations (grey) remains unknown in the present study. Finally, compared to LPFC, the ACC had a greater density of axonal targeting VGAT^+^ m2^+^ cartridges, presumably from PV^+^ chandelier cells.

Two functionally-distinct prefrontal regions involved in executive control, the LPFC and the ACC (Rushworth et al., 2011), differ in their structural relationship with the cholinergic modulatory system (Mesulam et al., 1992; Ghashghaei and Barbas, 2001). Figure 10 summarizes the normalized density of excitatory and inhibitory neuron types expressing mAChRs (Figure 10A) and the subcellular distribution of m1 and m2 receptors on a L3 pyramidal neuron and on the distinct inhibitory neuron subtypes with specific somatodendritic and axonal targets (Figure 10B) in these two prefrontal areas.

### Regional Differences in the Expression of mAChR on Subpopulations of Pyramidal Neurons

Our data revealed regional and laminar differences in the densities of excitatory subpopulations expressing m1^+^ or m2^+^. Consistent with previous studies, we found that MAP2^+^ pyramidal neurons, and the subset labeled by SMI-32^+^, were denser in LPFC than ACC (Barbas et al., 2018). However, a novel finding showed a greater density of the subset of SMI-32^+^ pyramidal neurons co-expressing calcium-binding protein CB^+^ in the ACC L3, compared to the LPFC (Figure 10). While CB has been used to mark inhibitory neurons in the primate cortex (DeFelipe, 1997), previous work has identified a small subset of lightly-expressing CB^+^ pyramidal neurons (Hof and Nimchinsky, 1992; DeFelipe, 1997; Kondo et al., 1999; Dombrowski et al., 2001), particularly within the paralimbic cortices and the hippocampus (Seress et al., 1991; Wang et al., 2021). The enrichment of CB^+^ in CA1 pyramidal neurons is interesting in light of the robust degrees of calcium-dependent dendritic and synaptic plasticity in these cells (Molinari et al., 1996; Westerink et al., 2012), and the role of CB^+^ expressing neurons in memory formation (Dumas et al., 2004; Li et al., 2017). Pyramidal neurons expressing SMI-32^+^ have been identified as a selectively vulnerable population in Alzheimer’s disease and other neurodegenerative diseases (Morrison and Hof, 2002; Bussiere et al., 2003). Therefore, our finding of m1^+^ and m2^+^ expressing SMI-32^+^ CB^+^ pyramidal neurons within the ACC warrants further investigation to understand the role of cholinergic modulation in memory and plasticity, and its implications on developing therapies for neurodegenerative and neuropsychiatric diseases (Levey, 1996; Moran et al., 2019; Foster et al., 2021).

### Denser m1 Expression in MAP2 Dendrites and Somata of ACC Pyramidal Neurons

In the L1 neuropil, we found a greater expression of m1^+^ in LPFC compared to ACC. However, in L1-L2 of LPFC, only ~18–21% of m1^+^ were expressed on MAP2^+^ dendrites, which is lower than in ACC, where ~29–37% of m1^+^ were expressed on MAP2^+^ dendrites. This suggests that the net functional effect of m1^+^ activation on L1-L2 MAP2^+^ dendrites, which are predominantly distal apical dendrites (Peters and Sethares, 1991), is greater in ACC than LPFC. Indeed, although a higher number of MAP2^+^ m1^+^ neurons was found in LPFC, the density of m1^+^ within somatic and proximal apical dendritic compartments of *individual* MAP2^+^ L3 pyramidal neurons was greater in ACC than LPFC (Figure 10). The greater density of dendritic m1^+^ expression in ACC pyramidal neurons is interesting in light of the role that muscarinic receptors play in modulating spike-timing-dependent dendritic plasticity (Yamasaki et al., 2010) and long-term potentiation (LTP; Markram and Segal, 1990; Marino et al., 1998; Dennis et al., 2016). The differential distribution of m1^+^ receptors across laminar cell types and compartments in ACC and LPFC suggest distinct cholinergic modulation of specific circuits (Coppola et al., 2016; Disney and Higley, 2020). The greater expression of m1^+^ in LPFC L1, compared to ACC, is likely due to non-MAP2 cells and compartments (Caceres et al., 1984; Peters and Sethares, 1991), such as axons, glial cells (Dombrowski et al., 2001), or weakly-stained interneuron dendrites (Gabbott, 2016; Schuman et al., 2019) enriched in this layer. L1 receives cortico-cortical “feedback” inputs and diffuse thalamic inputs that are thought to be important for shaping task-relevant signals (Roland, 2002; Jones, 2009). Our data suggest that these L1 inputs targeting MAP2^+^ dendrites in ACC and non-MAP2^+^ cellular compartments in LPFC are more modulated by m1^+^ mAChR activation. Future studies are needed to assess the subcellular localization of m1 on these non-MAP2 cells and their relationship with extrinsic inputs in the upper layers.

### Presynaptic m2 Receptors Are Selectively Localized on Excitatory Axon Terminals in ACC and LPFC

Electron microscopic work in rhesus monkeys showed that presynaptic m2^+^ receptors were predominantly localized to glutamatergic boutons forming synapses on dendritic spines (Mrzljak et al., 1993). We observed that m2^+^ receptors largely colocalized with presynaptic excitatory VGLUT1^+^ and VGLUT2^+^ axon terminals, suggesting a capacity for cholinergic suppression of cortico-cortical and cortico-subcortical excitatory transmission in both ACC and LPFC (Mrzljak et al., 1998; Hasselmo and Sarter, 2011; Medalla and Barbas, 2012). However, in the ACC, m2^+^ receptors were predominantly expressed on VGLUT1^+^ than on VGLUT2^+^ terminals indicating greater modulation of cortico-cortical axon terminals, consistent with previous findings in the PFC (Medalla and Barbas, 2012), as well as in the visual cortex (Disney et al., 2006). Interestingly, VGLUT1^+^ m2^+^ boutons were denser in L2/L3 compared to L1 in ACC, while VGLUT2^+^m2^+^ boutons were denser in L2/L3 compared to L1 in LPFC. These data suggest laminar and pathway-specificity of m2^+^ modulation of inputs within each area (Medalla and Barbas, 2012), with L2/3 cortico-cortical inputs in ACC but L2/3 cortico-subcortical inputs in LPFC may be suppressed by ACh to a greater extent compared to L1 inputs. In studies of rodent piriform cortex (Hasselmo and Bower, 1992) and monkey visual cortices (Disney et al., 2006; Disney and Aoki, 2008), ACh is thought to selectively suppress intrinsic cortico-cortical recurrent excitatory connections, but enhance extrinsic “bottom-up” inputs (i.e., thalamic input) for signal selection. However, our current data suggest that the relative degree of ACh suppression of distinct “bottom-up” vs. “top-down” pathways is region-specific. Indeed, in the primate visual system, the degree of ACh “top down” attentional modulation differs across areas, which is more robust in primary visual cortex (V1) and LPFC frontal eye fields (FEF), compared to the visual middle temporal area (MT; Herrero et al., 2008, 2017; Thiele et al., 2012; Veith et al., 2021). Further, previous anatomical work in monkey LPFC (Medalla and Barbas, 2012) showed differences in presynaptic m2 expression on distinct “top-down” prefrontal pathways, with m2^+^ ACC to LPFC inputs more predominant than m2^+^ LPFC to LPFC recurrent connections. The VGLUT2^+^ axon terminals in LPFC L2/3 examined here include the subset of thalamic “bottom-up” inputs from the higher-order mediodorsal and motor ventral anterior nuclei (Zikopoulos and Barbas, 2007), while VGLUT1^+^ terminals in ACC L2/3 include the subset of dense limbic “top-down” cortico-cortical input from entorhinal and orbitofrontal cortex (Timbie and Barbas, 2014; Calderazzo et al., 2021). Our data suggest preferential m2-mediated ACh suppression of these specific pathways. Future studies incorporating functional, anatomical tract-tracing, and computational work are needed to examine and confirm these layer- and pathway-specific patterns of cholinergic presynaptic modulation mediated by m2, and by other muscarinic and nicotinic receptor subtypes.

### Differential Expression of m2 Receptors in Neurochemically Distinct Inhibitory Neurons

The diverse distributions of m1^+^ and m2^+^ expressing inhibitory interneuron subtypes targeting distinct pyramidal neuronal compartments can differentially influence the activity in ACC and LPFC (DeFelipe, 1997; Kubota et al., 2016). While we found that almost all (92–100%) interneuron subtypes examined expressed m1^+^ in both prefrontal areas, laminar and regional density differences were found. PV^+^ m1^+^ neurons were densest in LPFC L3, while CB^+^ m1^+^ and CR^+^m1^+^ neurons were densest in ACC L2. The proportion of m2^+^ expressing neurons in each inhibitory neuron subtype differed between areas, with ACC exhibiting a higher proportion of PV^+^ m2^+^, CB^+^ m2^+^, and CR^+^ m2^+^ in L2 and L3 compared to LPFC. Our results suggest that while all interneurons are capable of being activated by m1 muscarinic receptors, the ability for m2 mediated muscarinic suppression differs depending on subtype, region and layer (Figure 10).

### Regional Differences in m2 Presynaptic Localization on Inhibitory Inputs Targeting Distinct Subcellular Postsynaptic Compartments

Consistent with the findings on the proportion of m2 expressing inhibitory neurons, direct examination of inhibitory terminals targeting distinct pyramidal neuron compartments revealed greater densities of m2^+^ inhibitory terminals in the ACC compared to the LPFC. These data collectively suggest that m2^+^ mediated muscarinic suppression of inhibitory neuron neurotransmitter release is likely to be greater in ACC than in LPFC.

Our findings show that the densities of somatic VGAT^+^ m2^+^ inhibitory inputs on L3 MAP2^+^ and SMI-32^+^ pyramidal neurons were greater in ACC than LPFC. We previously found that in LPFC, the majority of perisomatic inhibition is mediated by PV^+^ interneurons, but in ACC, perisomatic inputs from non-PV^+^, including cholecystokinin (CCK^+^) expressing inhibitory neurons predominate (Medalla et al., 2017). Indeed, here we found that the density of PV^+^ m1^+^ and PV^+^ m2^+^ expressing interneurons was greater in LPFC than in ACC. Thus, while studies to confirm the neurochemical identity of m2^+^ inhibitory axon terminals are ongoing, our previous work (Medalla et al., 2017) and current data suggest that non-PV^+^ perisomatic inhibitory inputs are the likely predominant targets of m2^+^ mediated suppression in the ACC. Further, m2^+^VGAT^+^ inhibitory cartridges, presumably belonging to PV^+^ chandelier cells (ChC) which target the axon-initial segment (AIS) of pyramidal neurons (Somogyi, 1977; Inan et al., 2013), were also more prevalent in the ACC, compared to the LPFC (Figure 10). In the ACC, 93% of VGAT^+^ cartridges co-expressed m2^+^, a substantially greater percentage compared to the 63% present in the LPFC. Further, we observed that these VGAT^+^ cartridges were longer in the ACC, suggesting a greater number of release sites, compared to LPFC. Moreover, the density of VGAT^+^ m2^+^ labeled boutons along strongly expressing m2^+^^+^ cartridges was greater in ACC than in the LPFC. Taken together, our data suggest that m2^+^ modulation of proximal perisomatic and AIS inhibition of L3 pyramidal neurons is greater in ACC than LPFC. These differences in the morphology and neuromodulation of VGAT^+^ cartridges on the AIS have important implications on action potential initiation (Lewis et al., 2011; Inan et al., 2013), excitability (Szabadics et al., 2006; Glickfeld et al., 2009; Woodruff et al., 2011), plasticity and temporal dynamics in these two prefrontal areas (Grubb et al., 2011; Kole and Stuart, 2012). Previous work has directly measured the length of AIS length in monkey LPFC and reported differences in length and inhibitory bouton density across development (Fish et al., 2013). In human schizophrenic patients, inhibitory cartridges in LPFC have been found to decrease in density (Woo et al., 1998; Rocco et al., 2017). To the best of our knowledge, this is the first time regional differences in VGAT^+^ cartridge lengths have been reported in the monkey prefrontal cortex, which has important implications on the dynamics of cognitive circuitry and its dysfunction in disease.

The current study also found regional differences in dendritic inhibition, presumably governed by distinct cell types. In contrast to PV^+^ interneurons that are densest in LPFC L3, the m1^+^/m2^+^ expressing CB^+^ and CR^+^, mainly dendritic-targeting, interneuron subtypes (DeFelipe, 1997) are most prevalent in ACC L2. In the ACC, VGAT^+^ proximal dendritic inputs, as well as, presumably distal CB^+^ VGAT^+^ axon terminals had a greater proportion colocalized with m2^+^ compared to LPFC (as summarized in Figure 10). While future immunolabeling and super-resolution or electron microscopy are needed to validate these compartment-specific synapses, our data suggest distinct suppression of GABA release from proximal and distal inhibitory synapses (Hajos et al., 1998; Salgado et al., 2007) that can lead to diverse temporal dynamics and synchrony across neuronal compartments in ACC and LPFC.

### Implications on Prefrontal Network Cholinergic Neuromodulation

In primate LPFC, *in vivo* electrophysiological studies have found that ACh, through muscarinic activation, increased firing rates and enhanced attentional modulation in broad and narrow spiking cells (Dasilva et al., 2019). However, m1 activation mainly suppressed delay-related activity in a heterogenous set of neurons in LPFC during a working memory task, with overstimulation of m1 resulting in disruption of rule representation (Vijayraghavan et al., 2018). These diverse ACh effects on cell-specific firing likely reflect how task-related activity emerges from concerted activation of distinct excitatory or inhibitory cell types, which cannot be reliably distinguished *in vivo* (Lee et al., 2021) and likely differ across cortical areas. Here, we present evidence that m1 receptors are widely expressed in both excitatory and inhibitory cell types in LPFC, providing the anatomical substrate for these diverse functional effects. However, while, indeed, excitatory neurons outnumber inhibitory neurons in ACC and LPFC, the proportion expressing m1 is lower for excitatory pyramidal neurons (80–70%) compared to inhibitory neurons, with almost 100% expressing m1. The prevalence of m1 expression on inhibitory neurons in these two prefrontal areas is greater than the proportions previously observed in monkey visual areas (Disney et al., 2006; Disney and Aoki, 2008). Thus, our anatomical data predict that m1 overstimulation in ACC and LPFC can potentially alter excitatory:inhibitory neuronal activity ratio, and produce strong net inhibition (Vijayraghavan et al., 2018). Further, our current and previous data (Medalla et al., 2017) show that total inhibition is greater in ACC than LPFC, but this inhibition in ACC can be more robustly diminished *via* presynaptic m2 receptors. While future computational work is needed to understand the direct implications of these circuits, our data are consistent with the role of ACC signals in many flexible goal-directed behaviors (i.e., error- conflict- signaling and task switching) that require inhibition to be engaged or dis-engaged depending on the task at hand (Botvinick et al., 2004; Brown and Braver, 2008; Quilodran et al., 2008; Chudasama et al., 2013; Voloh et al., 2015; Kolling et al., 2016; Kawai et al., 2019; Kim and Sejnowski, 2021).

In the ACC, the inhibitory axon terminals with the most robust m2^+^ expression belong to non-PV^+^ dendritic and perisomatic targeting cells, and AIS targeting ChC cells, all of which are thought to exhibit slow inhibitory synaptic kinetics (Figure 10; Nusser et al., 1996; Nyiri et al., 2001; Lewis et al., 2011; Tremblay et al., 2016). In rodents, CCK^+^, basket cells and PV^+^ ChC synapses are thought to be associated with α2 GABAergic subunits (Nyiri et al., 2001; Klausberger et al., 2002; Rocco et al., 2017). In contrast, PV^+^ basket cell synapses on somata are associated with the α1 GABAergic subunit with faster kinetics (Klausberger et al., 2002; Cardin et al., 2009). Further, in rhesus monkeys, CB dendritic-targeting inhibitory neurons (DeFelipe et al., 1989), unlike PV interneurons, are non-fast spiking (Zaitsev et al., 2005), characterized by long membrane time constants and action potential durations (Kawaguchi and Kubota, 1998; Zaitsev et al., 2005). Thus, our data suggest that ACh can reduce specifically slow inhibitory currents, to a greater degree in ACC than LPFC (Hajos et al., 1998; Salgado et al., 2007; Szabo et al., 2010). In contrast, the high density of m1^+^ PV^+^ neurons coupled with the low density of m2^+^ on perisomatic terminals on L3 pyramidal neurons in LPFC, suggests that ACh can increase firing of presumably fast-spiking PV^+^ basket cells without concurrent downstream suppression of their GABAergic release (Figure 10). This hypothesis is consistent with recent physiological work in the rodent frontal cortex showing that carbachol increased excitatory drive and depolarized L3 PV^+^ basket cells *via* m1^+^ activation, but did not affect the activity of PV^+^ ChC (Tikhonova et al., 2018).

Our data revealed that while ~92–100% of all inhibitory interneurons examined expressed m1^+^ and thereby can be activated by ACh, each subclass can be concurrently subject to specific m2^+^ mediated suppression at the level of their termination sites to influence network synchrony and information flow (Figure 10; Hajos et al., 1998; Salgado et al., 2007; Szabo et al., 2010; Colangelo et al., 2019). Previous work in rodents shows that coordinated perisomatic inhibition and depolarization of dendrites can govern theta activation and long-term potentiation in hippocampal CA1 (Kamondi et al., 1998; Bezaire et al., 2016). Muscarinic agonists can either drive theta or gamma activity depending on concerted activation of distinct inhibitory neurons and selective suppression of compartment-specific inhibitory synapses in the rodent cortex and hippocampus (Chapman and Lacaille, 1999; Levesque and Avoli, 2018). Given the important role of inhibitory synaptic kinetics on network oscillations (Kopell et al., 2010; Cardin, 2018) our data suggest that the greater capacity for ACh suppression of slow inhibition in ACC and potentiation of fast inhibition in LPFC can have a net effect of synchronizing fast oscillatory dynamics within and between the two areas (Medalla et al., 2017). This hypothesis is consistent with *in vivo* work in monkeys showing the role of gamma coordination within and between ACC and LPFC in cognitive processing, especially during behavioral shifts and error trials (Quilodran et al., 2008; Rothe et al., 2011). More detailed structural, physiological, and computational experiments are currently underway to examine the cholinergic modulation of specific GABAergic cell types, the contribution of distinct muscarinic, as well as, nicotinic receptors, and the downstream effects on network dynamics.

In summary, the data presented here shows the laminar- and cell-type-specific expression of m1 and m2 muscarinic receptors within ACC and LPFC that may underlie distinct cholinergic modulation in these prefrontal areas. We found laminar specificity in m1^+^ dendritic expression, and in m2^+^ presynaptic localization on cortico-cortical (VGLUT1^+^) and sub-cortical inputs (VGLUT2^+^), suggesting differential cholinergic modulation of “top-down” vs. “bottom-up” inputs in the two areas (Corbetta and Shulman, 2002; Disney et al., 2006). Further, our data suggest greater compartment-specific m2^+^ suppression of GABA-release in ACC L3 pyramidal neurons than in LPFC (Hajos et al., 1998; Salgado et al., 2007; Szabo et al., 2010). The anatomical localization of muscarinic receptors on distinct ACC and LPFC micro-circuits shown here sheds light on the functional outcomes of prefrontal cholinergic modulation of excitatory and inhibitory balance in normal behavior and its disruption in neuropsychiatric and neurological conditions.

## Data Availability Statement

The original contributions presented in the study are included in the article, further inquiries can be directed to the corresponding author.

## Ethics Statement

The animal study was reviewed and approved by Boston University Institutional Animal Care and Use Committee.

## Author Contributions

AT designed and performed experiments, data gathering, and analysis. MM conceptualized and designed the study and experiments. AT and MM wrote the manuscript. All authors contributed to the article and approved the submitted version.

## Conflict of Interest

The authors declare that the research was conducted in the absence of any commercial or financial relationships that could be construed as a potential conflict of interest.

## Publisher’s Note

All claims expressed in this article are solely those of the authors and do not necessarily represent those of their affiliated organizations, or those of the publisher, the editors and the reviewers. Any product that may be evaluated in this article, or claim that may be made by its manufacturer, is not guaranteed or endorsed by the publisher.

## Footnotes

^1^ http://imagej.nih.gov/ij/

^2^ https://imagej.net/Fiji
